# Implications of Cranial Arterial Stenosis and Dolichoectasia for Cerebral Small-Vessel Disease Etiopathogenesis: Findings From a Prospective Mild Stroke Cohort

**DOI:** 10.1161/CIRCULATIONAHA.126.079493

**Published:** 2026-05-06

**Authors:** Fei Han, Una Clancy, Carmen Arteaga-Reyes, Michael J. Thrippleton, Maria Del C. Valdés Hernández, Daniela Jaime Garcia, Michael S. Stringer, Ellen Backhouse, Francesca M. Chappell, Yajun Cheng, Dillys Xiaodi Liu, Junfang Zhang, Angela C.C. Jochems, Eleni Sakka, Charlotte Jardine, Gayle Barclay, Donna McIntyre, Iona Hamilton, Rosalind Brown, Yi-Cheng Zhu, Fergus N. Doubal, Joanna M. Wardlaw

**Affiliations:** 1 Department of Neurology, Peking Union Medical College Hospital, Chinese Academy of Medical Sciences and Peking Union Medical College, Beijing, China (F.H., Y.-C.Z.).; 2 Centre for Clinical Brain Sciences, Department of Neuroimaging Sciences, University of Edinburgh, UK (U.C., C.A.-R., M.J.T., M.D.C.V.H., D.J.G., M.S.S., E.B., F.M.C., A.C.C.J., E.S., R.B., F.N.D., J.M.W.).; 3 UK Dementia Research Institute at the University of Edinburgh, Edinburgh Medical School (U.C., C.A.-R., M.J.T., M.D.C.V.H., D.J.G., M.S.S., E.B., F.M.C., A.C.C.J., E.S., R.B., F.N.D., J.M.W.).; 4 Department of Neurology, West China Hospital, Sichuan University, Chengdu (Y.C.).; 5 Department of Psychiatry and Behavioral Sciences, University of California, San Francisco (D.X.L.).; 6 Department of Neurology, Shanghai Sixth People’s Hospital Affiliated to Shanghai Jiao Tong University School of Medicine, China (J.F.).; 7 Edinburgh Imaging Facility, Royal Infirmary of Edinburgh, UK (C.J., G.B., D.M., I.H.).

**Keywords:** atherosclerosis, carotid stenosis, cerebral small vessel diseases, dilatation, stroke, lacunar

## Abstract

**BACKGROUND:**

Stenosis and dolichoectasia of cranial arteries likely reflect distinct mechanisms. Their contributions to lacunar stroke and cerebral small-vessel disease (cSVD) remain contentious. We investigated the associations of large-artery stenosis (LAS) and arterial widening with stroke subtype, cSVD markers, incident infarcts, and clinical outcomes.

**METHODS:**

We prospectively recruited patients with lacunar or mild nonlacunar stroke, with demographic, stroke-related, cognitive, functional, and magnetic resonance imaging (index and incident infarcts, cSVD markers) assessments at baseline and 1 year. LAS was defined as ≥50% intracranial or cervical artery stenosis; basilar artery dolichoectasia was defined by basilar artery diameter, bifurcation height, and lateral displacement; and intracranial carotid and middle cerebral artery diameters were also measured. Associations were estimated from multivariable logistic, linear, and proportional odds regression models adjusted for age, sex, and vascular risk factors. We further conducted a systematic literature review to synthesize evidence on relationships between large-artery pathology and cSVD.

**RESULTS:**

Among 229 patients (mean age, 65.9±11.1 years; 131 [57.2%] lacunar stroke), LAS and basilar artery dolichoectasia were present in 20.5% and 15.7%, respectively. After adjustment, LAS (odds ratio, 0.49 [95% CI, 0.23–0.99]) and the presence of any embolic source were associated with lower odds of lacunar versus non-lacunar stroke, and not with cSVD markers or incident infarcts. In contrast, basilar artery dolichoectasia was strongly associated with lacunar stroke (odds ratio, 4.67 [95% CI, 1.87–13.14]), higher cSVD scores (ordinal analysis; odds ratio, 2.57 [95% CI, 1.28–5.25]), incident infarcts (75% subcortical; odds ratio, 2.29 [95% CI, 1.01–5.14]), and greater progression of white matter hyperintensities over 1 year (β, 0.15 [95% CI, 0.01–0.29] per log_10_-transformed volume). Similar associations were observed for wider intracranial arteries. The systematic review supported these findings.

**CONCLUSIONS:**

cSVD, including lacunar stroke, was unrelated to LAS but strongly associated with dolichoectasia and wider arteries. These findings support a nonatheromatous, intrinsic microvascular pathology, particularly segmental arteriolar disorganization, as the principal mechanism of lacunar stroke and cSVD. Mechanism-specific diagnostic and therapeutic strategies are warranted.

**Clinical Perspective**

**What Is New?**

- Large-artery stenosis was unlikely to represent a causal mechanism for lacunar stroke and showed no association with cerebral small-vessel disease (cSVD) imaging markers.
- Dolichoectasia and intracranial arterial widening emerged as vascular phenotypes strongly associated with cSVD, including its progression and lacunar stroke subtype.

**What Are the Clinical Implications?**

- Distinct large-artery phenotypes have divergent etiopathological implications for cSVD.
- Our findings support a nonatheromatous, intrinsic microvascular pathology as the principal mechanism of lacunar stroke and cSVD.
- Mechanism-based therapeutic strategies for lacunar stroke and cSVD, moving beyond conventional approaches focused on atherosclerosis or cardioembolism, are warranted.

Cranial artery stenosis and dolichoectasia likely represent distinct forms of large-artery pathology. Large-artery stenosis (LAS) arises from focal luminal narrowing due to atherosclerotic plaque,^1^ whereas dolichoectasia is generally considered to reflect nonatherosclerotic dilatation and tortuosity.^2^ These conditions differ in morphology and are thought to involve distinct pathophysiological processes, with potentially different impacts on and clinical implications for downstream small vessels and stroke subtypes. Although associations between large-artery disease and cerebral small-vessel disease (cSVD) have been reported,^3–5^ most studies were cross-sectional and rarely investigated both stenosis and dolichoectasia within well-phenotyped stroke cohorts with longitudinal follow-up.

Lacunar strokes, accounting for 20% to 30% of ischemic strokes,^6^ are attributed mainly to intrinsic cSVD, particularly the “segmental arterial disorganization” described in Fisher’s^7^ seminal clinicopathological studies. In patients with hypertension with lacunar stroke, pathological studies have documented arteriolar expansion, mural cellular and fibrinoid infiltration with wall destruction, and luminal occlusion by fibrinoid material. However, the relative contributions of atherothromboembolism, cardioembolism, and intrinsic microarteriopathy remain debated. Approximately 15% of patients with lacunar stroke harbor potential embolic sources, including large-artery atheroma, but their causal relevance is uncertain.^8–11^

In this prospective study of lacunar stroke, with nonlacunar ischemic strokes of similar severity as comparators, we investigated how atheromatous stenosis, dolichoectasia, and arterial widening relate to stroke subtypes, cSVD and its progression, longitudinal incident infarcts, and clinical outcomes. By directly comparing these 2 large-artery phenotypes within the same population, we aimed to clarify their respective roles in cSVD and the pathogenesis of lacunar stroke, with potential implications for targeted management.

## Methods

### Data Availability

The data supporting the findings of this study are available within the article and Supplemental Material. All other deidentified participant data and the analytical methods can be made available on reasonable request to the corresponding author.

### Study Design and Participants

**Figure 1.**
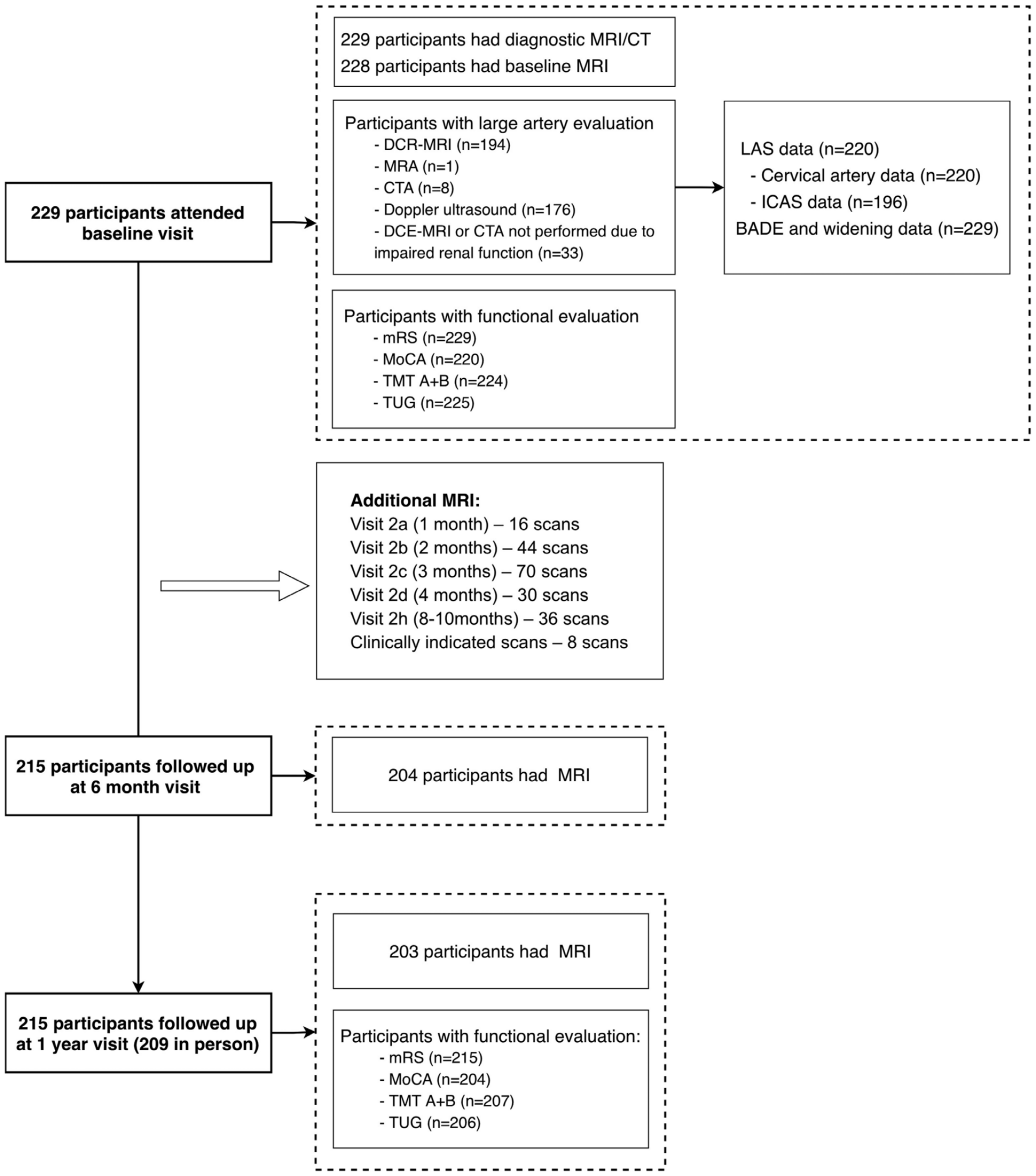
**Study flowchart.** BADE indicates basilar artery dolichoectasia; CTA, computed tomography angiography; DCE-MRI, dynamic contrast-enhanced magnetic resonance imaging; LAS, large-artery stenosis; MoCA, Montreal Cognitive Assessment; MRA, magnetic resonance angiography; mRS, modified Rankin Scale; TMT, Trail Making Test; and TUG, Timed Up-and-Go.

The Mild Stroke Study 3 is a prospective cohort of patients with recent lacunar or mild nonlacunar ischemic stroke consecutively recruited from Edinburgh stroke services between 2018 and 2021. The detailed protocol has been published.^12^ Mild stroke was defined as National Institutes of Health Stroke Scale score <8 at initial stroke presentation and modified Rankin Scale (mRS) score of ≤2 at enrollment (between 1 and 3 months after stroke). Exclusion criteria included contraindications to magnetic resonance imaging (MRI) and severe neurological, cardiac, or respiratory comorbidities. All underwent standard guideline-based stroke investigations and secondary prevention. Clinical and MRI assessments were conducted at baseline and at 6 and 12 months, with additional MRI scans at 3 or 9 months for those with lacunar stroke or moderate to severe white matter hyperintensity (WMH; Figure 1).

The study was approved by the Southeast Scotland Regional Ethics Committee (Ref.18/SS/0044), conducted under the Declaration of Helsinki, and registered under ISRCTN 12113543. All participants provided written informed consent.

### Clinical Measurements

The presenting (index) strokes were subtyped clinically as lacunar or nonlacunar stroke syndrome according to the Oxfordshire Community Stroke Project classification,^13^ with a corresponding recent infarct on diagnostic MRI or computed tomography or, if no visible infarct, no other explanation for the stroke symptoms. When clinical and imaging classifications differed, the imaging lesion defined the final subtype.^14^ Demographics, vascular risk factors, and cardiovascular comorbidities were recorded. Premorbid intelligence was assessed with the National Adult Reading Test.^15^ Functional status (mRS score), cognition (Montreal Cognitive Assessment [MoCA] score,^16^ Trail Making Test B/A ratio^17^), and mobility (Timed Up-and-Go score) were evaluated at baseline and 1 year. Recurrent stroke or transient ischemic attack was diagnosed with established criteria and confirmed by stroke specialists.

### Imaging Acquisition

All participants underwent diagnostic MRI or computed tomography at stroke presentation. Baseline and follow-up cerebrovascular imaging was performed with 1 dedicated 3-T research scanner (Siemens Prisma, Siemens Healthcare, Erlangen, Germany). Sequences included 3-dimensional T1-weighted, T2-weighted, fluid-attenuated inversion recovery, susceptibility-weighted, and diffusion-weighted imaging, with additional advanced protocols. At baseline, blood-brain barrier integrity was assessed with dynamic contrast-enhanced MRI with intravenous gadobutrol (0.1mmol/kg, Gadovist 1M; Bayer, Germany) unless estimated glomerular filtration rate was <30 mL/min. Detailed MRI parameters are provided in the Supplemental Methods and published protocol.^12,18^

### Imaging Analysis

All imaging analyses were performed by researchers blinded to clinical data and supervised by a neuroradiologist (J.M.W.). Structural MRI and large-artery assessments were conducted independently by different raters at separate time points, each blinded to the other.

#### Index and Incident Infarcts

Index and incident infarcts were defined as previously described.^18^ The index infarct was confirmed as an acute lesion on diagnostic imaging consistent with the presenting stroke syndrome. Radiologically detected incident infarcts were new infarcts between index stroke and 1-year follow-up that were visible on diffusion-weighted imaging, fluid-attenuated inversion recovery, or T2-weighted sequences and absent on prior scans. All infarcts were visually assessed on each diagnostic and study scan with a standardized proforma.^18^ Lesions were classified as subcortical or cortical; subcortical infarcts were further categorized by shape as round/ovoid or tubular.^19^

#### cSVD Markers

cSVD markers were assessed according to STRIVE-2 (Standards for Reporting Vascular Changes on Neuroimaging 2) criteria.^19^ Lacunes and microbleeds were counted, and a summary cSVD score was constructed.^20^ Quantitative MRI analyses applied semiautomated pipelines to segment and quantify intracranial volume, normal-appearing white matter, WMHs, perivascular spaces (PVSs), and infarct volumes.^21,22^ WMH progression was defined as baseline normal-appearing white matter converting to WMH at 1 year. Brain volume, WMH, and infarct volumes were normalized to intracranial volume (percent intracranial volume), whereas basal ganglia and centrum semiovale PVSs were normalized to the corresponding volumes of region of interest.

#### LAS and Dolichoectasia

Because magnetic resonance angiography was not routinely performed, intracranial atherosclerotic stenosis (ICAS) was assessed on arterial-phase dynamic contrast-enhanced MRI, reviewed in multiple planes, and graded at the narrowest lumen with established criteria (Figure S1).^23^ Intracranial arteries assessed included internal carotid arteries (ICAs), middle cerebral arteries (MCAs), anterior and posterior cerebral arteries, vertebral arteries, and basilar artery (BA). When stenosis was suspected, structural sequences were reviewed for confirmation. Cervical internal carotid artery (ICA) stenosis was further assessed by ultrasound and graded by the North American Symptomatic Carotid Endarterectomy Trial criteria.^24^ When available, magnetic resonance or computed tomography angiography was also reviewed. ICAS was defined as ≥50% stenosis in any intracranial artery; LAS was defined as ICAS or ≥50% cervical artery stenosis. A potential embolic source included LAS or major cardiac abnormalities (atrial fibrillation, patent foramen ovale, ischemic heart disease, valvular disease, or heart failure).

Maximum diameters of the BA, bilateral ICAs (vertical cavernous segment), and MCAs (M1 segment) were measured on axial T2-weighted images to 0.01 mm (Figure S2). In stenotic cases, the least affected segment was measured. Diameters were adjusted for intracranial volume with residual correction, and bilateral ICA/MCA diameters were averaged. BA bifurcation height and lateral displacement were rated with a validated four-point scale (Figure S2).^25^ Bifurcation height was scored as follows: 0=at/below dorsum sellae, 1=within suprasellar cistern, 2=at the level of third ventricle floor, or 3=indenting and elevating third ventricle floor. Lateral displacement was graded by maximal lateral deviation: 0=midline, 1=medial to clivus or dorsum sellae margin, 2=lateral to this margin, or 3=cerebellopontine angle cistern. BA dolichoectasia (BADE) was defined as BA diameter >4.5 mm, bifurcation height ≥2, or lateral displacement ≥2.^25^

Intrarater reliability showed that the intraclass correlation coefficients for diameters were 0.95 (BA), 0.85 (ICA), and 0.9 (MCA). Weighted κ was 0.81 for ICAS and 0.85 for BADE.

### Systematic Review

We systematically reviewed studies investigating associations between large-artery pathology and lacunar stroke or cSVD. MEDLINE was searched through June 22, 2025. Random-effects meta-analyses were performed to estimate the prevalence and laterality of LAS in lacunar stroke. The full search strategy, selection criteria, and methodological details are provided in the Supplemental Methods.

### Statistical Analysis

Data are presented as mean±SD, median (interquartile range), or count (percent). Group comparisons used χ^2^ tests for categorical and *t* or Mann-Whitney *U* tests for continuous variables. Infarct analyses were conducted at both the individual (per-person) and lesion (per-infarct) levels. At the individual level, multivariable regression models were constructed to assess associations of LAS and BADE with index stroke subtype, number and volume of index infarcts, incident infarcts, and recurrent stroke or transient ischemic attack. Covariates were specified a priori from established vascular risk factors, prior epidemiological studies of cerebrovascular outcomes, and consistent adjustment strategies used in our previous work^18^ to minimize residual confounding. Models were adjusted for age, sex, smoking, hypertension, diabetes, hyperlipidemia, and body mass index. Variance inflation factors were all <1.3, indicating no evidence of multicollinearity. At the lesion level, Rao-Scott cluster-adjusted χ^2^ tests were used to account for multiple infarcts per patient when infarct subtype with LAS, BADE, or embolic sources were compared. Among patients with LAS, infarct location relative to the stenotic artery was also examined.

Associations of LAS and BADE with cSVD markers at baseline and 1 year were analyzed with multivariable linear regression for continuous outcomes and ordinal logistic regression for ordinal outcome (summary cSVD score). The proportional odds assumption for ordinal models was verified by the Brant test. Models were adjusted for age, sex, and vascular risk factors. WMH and PVS volumes were log_10_ transformed. Lacune count was included in models of brain volume, and 1-year outcomes were additionally adjusted for baseline cSVD score. Results are reported as odds ratios (ORs) or regression coefficients with 95% CIs.

Clinical outcomes were analyzed with ordinal logistic regression for mRS score and linear regression for continuous measures (MoCA score, Trail Making Test B/A ratio, and Timed Up-and-Go score). The proportional odds assumption for ordinal models was verified by the Brant test. All models were adjusted for age, sex, and baseline cSVD score, with additional adjustment for National Adult Reading Test score in cognitive measures (MoCA score and Trail Making Test B/A ratio).

Sensitivity analyses tested the robustness of the main findings in index and incident infarct models by (1) examining ICAS, any potential embolic source, and atrial fibrillation instead of LAS and (2) adjusting for baseline cSVD score. For analyses focusing on cSVD markers, sensitivity analyses further included (3) examining ICAS, any potential embolic source, and atrial fibrillation instead of LAS and substituting BADE with continuous arterial diameters (ICA, MCA, and BA). In models in which arterial diameters were analyzed as continuous exposures, LAS status was included as an additional covariate. (4) Statistical interaction terms between LAS and BADE were included in the aforementioned models to assess potential effect modification. All analyses were performed with R (version 4.4.2), with 2-sided values of *P*<0.05 considered significant.

## Results

### Baseline Characteristics

We recruited 229 participants (mean age, 65.9±11.1 years; 152 [66.4%] male; Table 1), including 131 (57.2%) with lacunar stroke and 98 (42.8%) with nonlacunar stroke. Most participants (n=168) had a single index infarct; 25 had 2; and 20 had ≥3. LAS was identified in 47 participants (20.5%): 40 (17.4%) with ICAS, 13 with cervical ICA stenosis, and 6 with both. A potential embolic source was present in 97 participants (42.4%), of whom 23 had atrial fibrillation, and BADE was present in 36 (15.7%). Details of stenosis location and arterial diameters are presented in Tables S1 and S2. At baseline, 204 participants (89.1%) received antiplatelet therapy, 26 (11.4%) received anticoagulation, and 211 (92.1%) were treated with lipid-lowering medications.

**Table 1.**
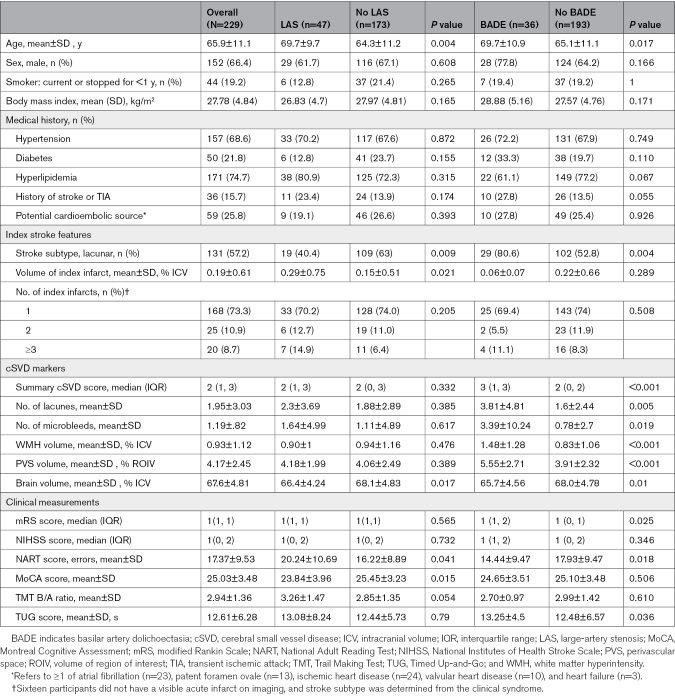
Baseline Characteristics of the Study Population

### Associations With Index Stroke

**Figure 2.**
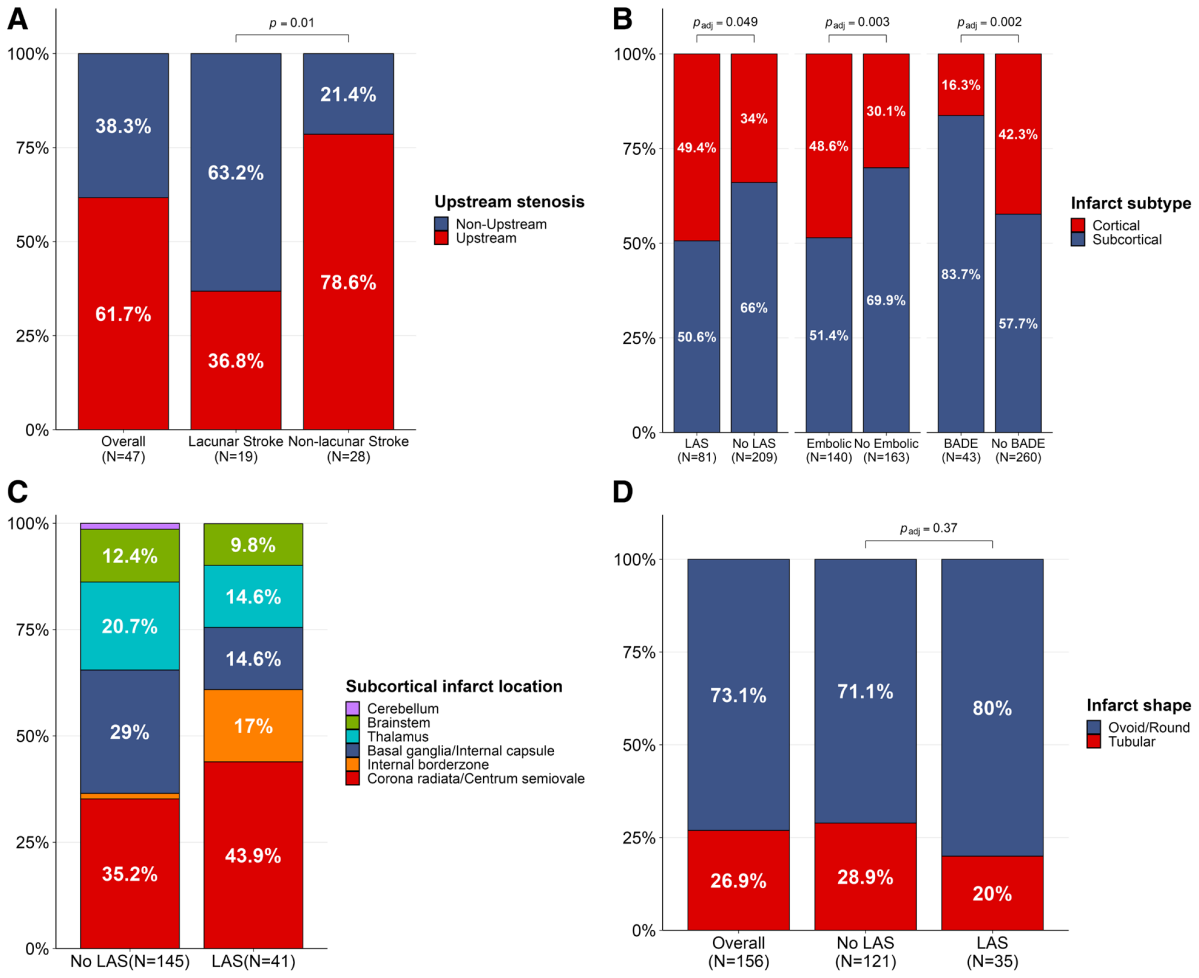
**Distributions and anatomical features of index infarcts. A**, Territorial concordance between index strokes and upstream stenosis in the per-person analysis among 47 patients with large-artery stenosis (LAS). **B**, Distribution of 303 infarcts at index stroke in the per-infarct analysis, stratified by the presence or absence of LAS, any potential embolic source, and basilar artery dolichoectasia (*P* values from Rao-Scott cluster-adjusted χ^2^ tests). **C**, Distribution of anatomical locations of 186 subcortical infarcts at index stroke in the per-infarct analysis, stratified by the presence of LAS. **D**, Distribution of shapes of subcortical infarcts in the per-infarct analysis, stratified by the presence of LAS (*P* value from Rao-Scott cluster-adjusted χ^2^ tests).

LAS was more prevalent in nonlacunar than lacunar stroke (28/98 [28.5%] versus 19/131 [14.5%]). Among patients with LAS, most had nonlacunar strokes (22/28, 78.6%), but only 36.8% of lacunar strokes (7/19) involved the territory of the stenotic artery (*P*=0.01; Figure 2A). After adjustment for age, sex, and vascular risk factors, LAS remained less frequent in lacunar stroke (OR, 0.49 [95% CI, 0.23–0.99]) and was associated with a greater number of index infarcts (β, 0.54 [95% CI, 0.14–0.93]), whereas its correlation with infarct volume was not retained (Table 2). BADE was more frequent in lacunar than nonlacunar stroke (29/131 [22.1%] versus 7/98 [7.1%]) and remained independently associated with higher odds of lacunar stroke after adjustment (OR, 4.67 [95% CI, 1.87–13.14]).

**Table 2.**
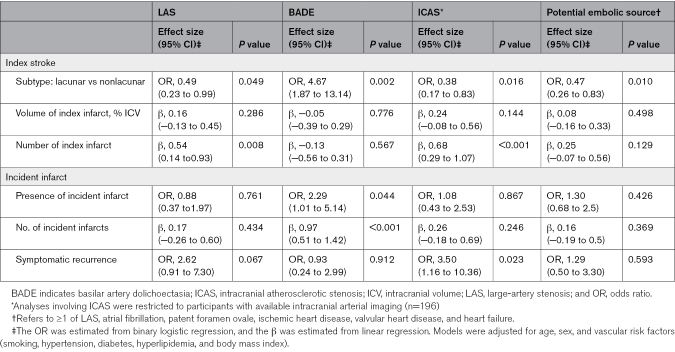
Multivariable Regression Analyses of Associations Between LAS, BADE, ICAS, and Any Potential Embolic Source With Index Stroke and Incident Infarcts

In the per-lesion analysis of 303 index infarcts (186 [61.4%] subcortical, 117 [38.6%] cortical; Table S3), cortical infarcts were more frequent with LAS (49.4% versus 34%, *P*=0.049) and potential embolic source, whereas subcortical infarcts were more frequent with BADE (83.7% versus 57.7%; *P*=0.002; Figure 2B). With LAS, subcortical infarcts more frequently involved corona radiata, centrum semiovale, and internal border zone, whereas without LAS, they more frequently affected basal ganglia, thalamus, and brainstem (Figure 2C). Infarct shape did not differ by LAS (Figure 2D). Posterior circulation infarcts did not differ by BADE, but brainstem involvement was more frequent with BADE (16.3% versus 5.8%; Table S4).

### Associations With Incident Infarcts

Over 1 year, 130 incident infarcts (97 [74.6%] subcortical, 33 [25.4%] cortical) occurred in 59 participants. At the individual level, BADE was associated with higher odds of incident infarcts (OR, 2.29 [95% CI, 1.01–5.14]) and a greater infarct count (β, 0.97 [95% CI, 0.51–1.42]) after adjustment, whereas LAS showed no significant association (Table 2). Recurrent symptomatic stroke or transient ischemic attack occurred in 22 participants, with a significant association with ICAS (OR, 3.50 [95% CI, 1.16–10.36]; *P*=0.023).

At the lesion level, cortical incident infarcts were more frequent with an embolic source (36.8% versus 16.4%), whereas subcortical incident infarcts were more frequent with BADE (86% versus 67.5%). Among 26 incident infarcts in 10 patients with LAS, all cortical infarcts (5/5) occurred downstream of the stenosis, whereas most subcortical infarcts (15/21; *P*=0.049) did not. Subcortical infarcts involved predominantly cerebral white matter (Figure S3 and Table S5).

### Associations With cSVD Markers and Progression

The proportional odds assumption was satisfied for the ordinal cSVD score in both models (Brant test, *P*=0.35 for the LAS model and 0.16 for BADE model). After adjustment for demographics and vascular risk factors, LAS was not associated with baseline cSVD markers (Table 3). In contrast, BADE was associated with multiple markers, including more lacunes (β, 2.31 [95% CI, 1.27–3.36]) and microbleeds (β, 3.41 [95% CI, 1.68–5.13]), greater WMH (β, 0.26 [95% CI, 0.11–0.41]) and PVS (β, 0.10 [95% CI, 0.02–0.18]) volumes, and higher summary cSVD score (OR, 2.57 [95% CI, 1.28–5.25]). No associations were observed between BADE and brain volume. At 1 year, BADE remained associated with all cSVD markers and with WMH progression (β, 0.15 [95% CI, 0.01–0.29]), whereas LAS was associated only with more lacunes.

**Table 3.**
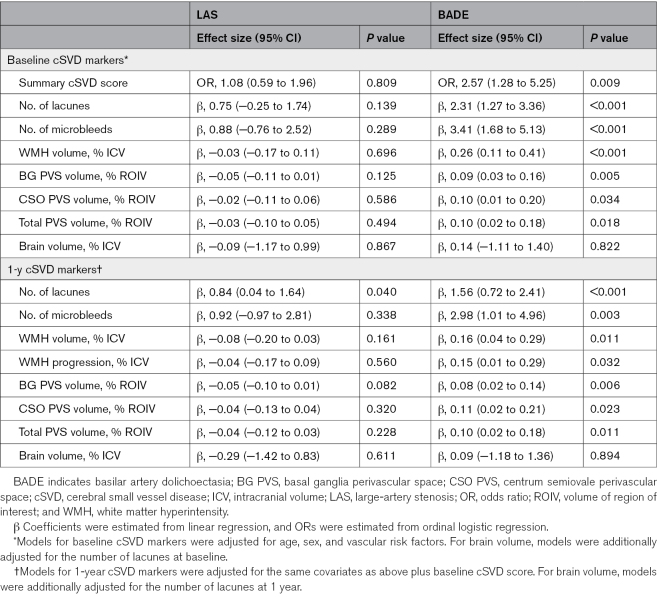
Multivariable Regression Analyses of Associations Between LAS, BADE, and cSVD Markers at Baseline and 1 Year

### Associations With Clinical Outcomes

In univariate analyses, LAS was associated with lower MoCA scores, and BADE was associated with higher mRS score and longer Timed Up-and-Go times (Table 1). The proportional odds assumption was met for mRS score in both models (Brant test, *P*=0.42 for LAS and 0.36 for BADE). After adjustment, neither LAS nor BADE remained significantly associated with functional (mRS score), cognitive (MoCA score, Trail Making Test B/A ratio), or mobility (Timed Up-and-Go score) outcomes at baseline or 1 year (Table 4).

**Table 4.**
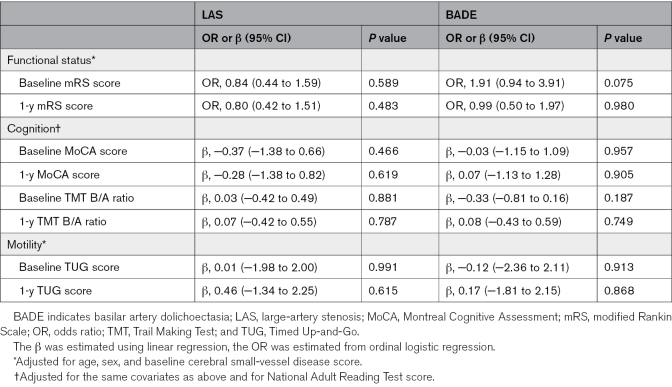
Associations Between LAS, BADE, and Clinical Outcomes

### Sensitivity Analysis

Associations of ICAS and potential embolic source with index and incident infarcts were consistent with those of LAS (Table 2). Additional adjustment for baseline cSVD score did not alter these associations (Table S6). In models examining cSVD markers, results remained broadly consistent when BADE was modeled with continuous arterial diameters and when LAS was replaced by ICAS, potential embolic source, or atrial fibrillation (Table S7). In addition, inclusion of an interaction term between LAS and BADE in both the infarct and cSVD models did not indicate evidence of effect modification (Table S8).

### Systematic Review

Findings from the systematic review are detailed in the Supplemental Material (Tables S9–S16 and Figures S4-S7). Across 27 studies comprising 9515 patients with lacunar stroke, the pooled prevalence of ipsilateral LAS was 11% (95% CI, 8%–15%; Table S9 and Figure S5A–S5C), higher in Asian cohorts than White cohorts (19% versus 9%; Figure S5D and S5E). In 13 studies directly comparing ipsilateral and contralateral LAS, no significant difference was found (pooled risk difference, 0.01 [95% CI, −0.04 to 0.06]; Figure S5F), providing little evidence for a causal relationship. In community cohorts, LAS was often correlated with lacunes and WMH, but findings in stroke cohorts were inconsistent (Tables S10–S13). Even when associations existed, no consistent laterality was observed between LAS and lacunes or WMH (Table S11 and S13). Most studies reported no association between LAS and microbleeds (Table S14) or PVS (Table S15). Fifteen cross-sectional studies assessing dolichoectasia showed associations with lacunar stroke and multiple cSVD markers, which were further supported by a meta-analysis (Table S16 and Figure S7).

## Discussion

In this prospective cohort of patients with lacunar stroke enriched for cSVD features and nonlacunar controls, cranial artery stenosis and dolichoectasia showed distinct associations with stroke subtype, cSVD, and cSVD progression. LAS was associated with nonlacunar stroke but was uncommon in lacunar stroke and not associated with MRI-defined incident infarcts or cSVD markers. Similar patterns were observed for ICAS and embolic source. In contrast, BADE was more frequent in lacunar subtype and, together with larger arterial diameters, was strongly associated with all cSVD markers except atrophy. BADE was also associated with more incident infarcts on follow-up MRI, most of which were subcortical, but not with symptomatic recurrence. Neither LAS nor BADE independently predicted functional, cognitive, or mobility outcomes at baseline or 1 year.

The prevalence of LAS (20.5%) in our cohort was comparable to previous reports in minor stroke.^26,27^ LAS and other embolic sources were more frequently associated with nonlacunar than lacunar stroke, across both index and incident infarcts and in per-person and per-lesion analyses. Although nonlacunar strokes often aligned with proximal stenosis, lacunar strokes showed weaker territorial concordance, suggesting noncausal coexistence. This is supported by the Secondary Prevention of Small Subcortical Strokes trial, in which 17.3% of patients with lacunar stroke had ICAS but only 1.8% had relevant ipsilateral stenosis.^28,29^ Similarly, our meta-analysis demonstrated no difference in stenosis prevalence between ipsilateral and contralateral arteries in lacunar stroke. These findings are consistent with Fisher’s^7^ seminal pathological observations. Although all 4 patients that Fisher examined had severe atherosclerosis at the patient level, reflecting shared vascular risk factors, lesion-level analysis showed that the majority of lacunar lesions were attributable to intrinsic small-vessel disease, called segmental arterial disorganization (40/50 [80%]), whereas parent artery atheroma directly occluding the arteriole supplying the lacunar lesion was rare (3/50 [6%]).^7^

In patients with LAS, lacunar-appearing infarcts were more often located in internal border zone rather than deep perforator territories and were frequently multiple. This pattern supports that proximal stenosis may contribute through embolic or hemodynamic mechanisms, particularly when infarcts are multifocal and unilateral, rather than by direct perforator occlusion.^11^ These findings highlight the need for individualized etiologic assessment. In typical lacunar stroke, in which intrinsic cSVD predominates, nonselective embolic screening may have limited diagnostic value, although it remains essential to exclude treatable large-artery or cardioembolic causes when suspected.

Although ICAS was associated with symptomatic recurrent stroke or transient ischemic attack, consistent with previous studies,^26,30^ it was unrelated to new MRI-detected infarcts, which were mostly covert, small, and subcortical. More than one-quarter of participants developed these incident infarcts despite guideline-based secondary stroke prevention, and they were strongly associated with baseline cSVD burden,^18^ supporting cSVD as a progressive, largely nonatheromatous process. Despite the presence of shared vascular risk factors across stroke subtypes, our findings suggest distinct downstream pathophysiological mechanisms, with atherosclerosis predominating in nonlacunar stroke and intrinsic microvascular disease underlying lacunar (cSVD-related) stroke. Current secondary prevention strategies for lacunar stroke and cSVD largely mirror those used for large-artery atherosclerotic disease, including vascular risk factor control and antihypertensive, antiplatelet, and lipid-lowering treatment. Although these approaches remain important and should not be discounted, accumulating evidence, consistent with our results, suggests that guideline-based secondary stroke prevention, including antiplatelet therapy and statins, has limited efficacy in preventing the progression of cSVD-related brain damage.^31,32^ Collectively, these findings emphasize that in lacunar stroke with substantial cSVD burden, management should target microvascular pathology, for example, through therapies aimed at improving microvascular function, rather than relying solely on atherosclerosis-focused approaches.^33–36^

Although some community-based studies have reported associations between LAS and certain cSVD markers, findings in stroke populations were inconsistent. In our mild stroke cohort, LAS showed no such association. This discrepancy may reflect methodological differences: Inclusion of a well-defined nonlacunar control group and rigorous adjustment for vascular risk factors and baseline cSVD enabled distinction between coassociation and causation. Many prior studies lacked appropriate controls, inadequately adjusted for confounders, and included heterogeneous populations or disease stages, likely explaining the inconsistent results.

Consistent with previous studies,^4^ BADE was strongly associated with multiple cSVD features, including a 4-fold increased odds of lacunar stroke, greater cSVD burden and progression, and more incident subcortical infarcts. Several mechanisms may underlie this association. First, shared genetic susceptibility may contribute: Mutations in basement membrane protein genes (eg, *COL4A1* and *COL4A2*) have been identified in patients with dolichoectasia and cSVD and have shown associations with WMH in genome-wide association studies.^37,38^ In addition, studies of cerebral autosomal dominant arteriopathy with subcortical infarcts and leukoencephalopathy, a monogenic form of cSVD caused by *NOTCH3* mutations, have demonstrated dilatation and tortuosity of large intracranial arteries in both human histopathological studies and experimental models.^39,40^ These findings support a genetic continuum linking large-artery widening and intrinsic small-vessel arteriopathy. Second, both conditions may reflect connective tissue abnormalities of the vessel wall. Fisher^7^ and others^41–43^ described microarteriolar dilatation, elastic lamina fragmentation, and medial rarefaction in cSVD, possibly due to extracellular matrix imbalance and dysregulated metalloproteinase activity, leading to arterial weakening and widening in large vessels and segmental disorganization in small arterioles. Third, widened or tortuous arteries may exert mechanical stress on perforating arterioles, disrupting local flow and promoting microvascular injury.^44^ Supporting this, we observed more pontine infarcts in BADE.

To the best of our knowledge, this is the first study to evaluate 2 distinct large-artery pathologies within a single, well-characterized cohort, controlling for stroke subtype and medication, enabling direct comparison of their associations with stroke subtypes and cSVD. Key strengths include its prospective design, standardized and volumetric assessment of comprehensive cSVD features at both patient and lesion levels, and blinded evaluation of large-artery characteristics to all clinical, imaging, and outcome data. The findings were further contextualized within an updated systematic review and meta-analysis. Several limitations should be acknowledged. First, ICAS was evaluated with arterial-phase dynamic contrast-enhanced MRI rather than dedicated angiography. Although potentially less sensitive to mild stenosis, the slow-injection technique provides high-quality luminal visualization comparable to conventional bolus magnetic resonance angiography, making it unlikely to miss moderate or severe stenosis. Second, the lack of vessel wall imaging limited direct visualization of intramural plaque at perforator origins. Third, the single-center sample limits generalizability, underscoring the need for larger multicenter studies to validate and extend these findings.

## Conclusions

Large-artery pathologies exhibit divergent etiopathological implications for stroke subtype and cSVD. Dolichoectasia reflects a cSVD-related phenotype, whereas LAS was uncommon in lacunar stroke and unrelated to cSVD markers. These findings support that lacunar stroke and other cSVD manifestations are driven predominantly by intrinsic, nonatheromatous microvascular pathology, highlighting the need for mechanism-based diagnostic and therapeutic strategies beyond conventional approaches targeting atheroma or cardioembolism.

## Article Information

### Acknowledgments

The authors thank the staff and participants of the Mild Stroke Study 3 for their important contributions.

### Disclosures

None.

### Supplemental Material

Supplemental Methods

Tables S1–S16

Figures S1–S7

STROBE checklist

References 45–127

## Supplementary Material
