# Supplementary material for: Implications of Cranial Arterial Stenosis and Dolichoectasia for Cerebral Small-Vessel Disease Etiopathogenesis: Findings From a Prospective Mild Stroke Cohort
Source: Circulation. 2026 May 6;153(23):1813–26. doi: 10.1161/CIRCULATIONAHA.126.079493 (PMC13236046; doi:10.1161/CIRCULATIONAHA.126.079493)
Supplement: Supplementary file 1 [file cir-153-1813-s001.pdf]

Implications of cranial arterial stenosis and dolichoectasia for cerebral small vessel disease etiopathogenesis: findings from a prospective mild stroke cohort

SUPPLEMENTAL MATERIAL

Supplemental Methods
1. Definition of vascular risk factors
2. MRI protocol
3. Systematic review and meta-analysis

Supplemental Tables
Table S1. Distribution and severity of stenosis across intracranial and cervical arteries
Table S2. Distribution of intracranial artery diameters and basilar artery morphology
Table S3. Anatomical distribution of 303 index infarcts in the per-infarct analysis
Table S4. Distribution of posterior circulation index infarcts in the per-infarct analysis, stratified by presence or absence of BADE
Table S5. Anatomical distribution of 130 incident infarcts in the per-infarct analysis
Table S6. Sensitivity analyses: associations of large artery characteristics and embolic source with index stroke and incident infarcts, with additional adjustment for baseline cSVD score
Table S7. Sensitivity analysis: associations between ICAS, embolic source, arterial diameters and cSVD markers
Table S8. Sensitivity analysis: models including LAS×BADE interaction for index stroke, incident infarcts, and cSVD markers
Table S9. Comparison of ipsilateral and contralateral LAS in lacunar stroke
Table S10. Studies evaluating the association between LAS and SBI/lacunes
Table S11. Studies evaluating the laterality of SBI/lacunes by the side of LAS
Table S12. Studies evaluating the association between LAS and WMH
Table S13. Studies evaluating the lateral association between LAS and WMH
Table S14. Studies evaluating the association between LAS and CMB
Table S15. Studies evaluating the association between LAS and PVS
Table S16. Studies evaluating the association between intracranial artery dolichoectasia and cSVD

Supplemental Figures and Figure Legends
Figure S1. Illustration of severe stenosis in the left middle cerebral artery
Figure S2. Assessment of intracranial artery dolichoectasia
Figure S3. Distributions and anatomical features of incident infarcts
Figure S4. PRISMA diagram for systematic review
Figure S5. Meta-analysis of the prevalence of large artery stenosis in patients with lacunar stroke
Figure S6. Sensitivity analyses of the pooled prevalence and risk difference estimates after exclusion of the study reporting the lowest prevalence.
Figure S7. Meta-analysis of the association between dolichoectasia and cerebral small vessel disease

STROBE Checklist

Supplemental Methods

1. Definition of vascular risk factors

Risk factors	Definition
Smoking	Current smoking or cessation within the previous 12 months
Body mass index	Weight in kilograms divided by height in meters squared (kg/m²)
Hypertension	(1) A documented clinical diagnosis of hypertension in the medical record; or<br>(2) Use of antihypertensive medication; or<br>(3) Systolic blood pressure ≥140mmHg or diastolic blood pressure ≥90mmHg on repeated measurements.
Diabetes mellitus	(1) A documented diagnosis of diabetes mellitus in the medical record; or<br>(2) Use of glucose-lowering medication (oral agents or insulin); or<br>(3) HbA1c ≥6.5% or fasting plasma glucose ≥7.0 mmol/L.
Hyperlipidemia	(1) A documented clinical diagnosis of hyperlipidemia; or<br>(2) Use of lipid-lowering therapy (e.g. statins); or<br>(3) Total cholesterol ≥5.0 mmol/L.

2. MRI protocol

2.1 Structural imaging included 3D T1-weighted, T2-weighted, fluid attenuated inversion recovery, susceptibility-weighted, and diffusion-weighted imaging.

2.2 Blood-brain barrier integrity was assessed using dynamic contrast-enhanced (DCE) MRI unless the estimated glomerular filtration rate was below 30 mL/min. DCE-MRI was acquired with a 3D sagittal T1-weighted spoiled gradient-echo sequence consisting of 32 volumes at a temporal resolution of 39.6 s. After three pre-contrast volumes, gadobutrol (0.1 mmol/kg body weight; 1M Gadovist, Bayer AG, Leverkusen, Germany) was administered intravenously over 110-130 s using a power injector, followed by a 20 mL saline flush.

	T1W	FLAIR	PD	T2W	SWI	DCE-MRI
Sequence	MPRAGE (3D IR-sGRE)	SPACE (3D RARE)	3D sGRE	SPACE (3D RARE)	3D sGRE	T1w 3D sGRE
Voxel size	1×1×1	1×1×1	1×1×1	0.9×0.9×0.9	0.6×0.6×3	2×2×2
TR (ms)	2500	5000	6.04	3200	28	3.44
TE (ms)	4.37	388	2.44	408	20	1.68
TI	-	1100	1800	-	-	-
Flip Angle	7°	-	2°	-	9°	15°
Acquisition time (m:s)	3:45	5:57	1:57	3:42	4:02	21:08
Other	R=3	R=3	R=3	R=2×2	R=2	32 volumes

3. Systematic review and meta-analysis

3.1 Search strategy and selection criteria

We searched MEDLINE (via PubMed) from database inception to June 22, 2025. The full search strategies for each research question are provided below. Search terms combined synonyms for lacunar stroke and cSVD imaging markers (lacunes, WMH, CMB, and PVS) with terms related to large artery pathology (stenosis, intracranial atherosclerosis, and dolichoectasia). No restrictions were applied on

study design or publication date, but only human studies published in English were considered.

Studies were eligible if they were observational cohorts, cross-sectional studies, or relevant arms of randomized controlled trials, included at least 50 participants, and reported data on large artery stenosis or dolichoectasia in relation to lacunar stroke or cSVD imaging markers. We excluded case reports, autopsy series, studies without relevant imaging outcomes, and interventional or surgical trial arms.

**MEDLINE was systematically searched using the following strategy**<br>**From inception to 22 June 2025**
Question: Association between large artery stenosis and side-specific occurrence of lacunar stroke<br>((lacunar[tiab] OR subcortical[tiab]) AND (stroke[tiab] OR infarct*[tiab] OR infarction[tiab])) AND (stenosis[tiab] OR stenoses[tiab] OR "large artery disease"[tiab] OR carotid[tiab] OR atherosclerosis[tiab] OR atherosclerotic[tiab]) AND english[lang]
Question: Association between large artery stenosis and cerebral small vessel disease (silent brain infarcts/acunes, white matter lesions, cerebral microbleeds and perivascular spaces)<br>(Lacune[tiab] OR (silent[tiab] AND (stroke[tiab] OR infarct*[tiab] OR infarction[tiab])) OR "White matter lesion"[tiab] OR "White matter hyperintensity"[tiab] OR "White matter hyperintensities"[tiab] OR"leukoaraiosis"[tiab] OR Microbleed[tiab] OR "Perivascular space"[tiab] OR "Virchow-Robin space"[tiab] OR "Small vessel disease"[tiab] OR "Small vessel diseases"[tiab]) AND (Stenosis[tiab] OR Stenoses[tiab] OR Stenotic[tiab] OR Atherosclerosis[tiab] OR Atherosclerotic[tiab]) AND ("Cerebral artery"[tiab] OR Carotid[tiab] OR "Intracranial"[tiab] OR "Large artery"[tiab]) AND english[lang]
Question: Association between dolichoectasia and cerebral small vessel disease<br>(lacune[tiab] OR lacunar[tiab] OR "white matter lesion"[tiab] OR "white matter lesions"[tiab] OR "white matter hyperintensity"[tiab] OR "white matter hyperintensities"[tiab] OR microbleed[tiab] OR microbleeds[tiab] OR "perivascular space"[tiab] OR "perivascular spaces"[tiab] OR "virchow-robin space"[tiab] OR "virchow-robin spaces"[tiab] OR "small vessel disease"[tiab] OR "small vessel diseases"[tiab]) AND (dolichoectasia[tiab] OR dolichoectatic[tiab] OR dilatation[tiab] OR elongation[tiab] OR tortuosity[tiab]) AND ("cerebral"[tiab] OR basilar[tiab] OR vertebrobasilar[tiab] OR "intracranial"[tiab] OR "arterial"[tiab]) AND english[lang]

3.2 Study selection and data extraction

A single comprehensive search was conducted using six prespecified search strategies addressing different research questions. After removing duplicates, titles and abstracts were screened, followed by full-text review. Studies were then allocated to one or more research questions depending on their reported outcomes. Data were extracted according to a structured template, including study design, country or region, sample size, population characteristics, imaging modality, definitions of large artery stenosis and dolichoectasia, and rating methods for cSVD markers. When available, effect estimates, or raw data were collected to enable calculation of prevalence, laterality, and associations. Discrepancies between reviewers were resolved through discussion, and the extracted data were synthesized in summary tables for both qualitative and quantitative analyses.

3.3 Statistical analysis

Random-effects meta-analyses were performed to estimate the pooled prevalence of ipsilateral large artery stenosis in patients with lacunar stroke. Proportions were pooled using logit transformation

(PLOGIT) with inverse-variance weighting and back-transformed for interpretation. To improve variance stability for studies with very low prevalences, a continuity correction (0.5) was applied when necessary. Between-study variance (τ^2) was estimated using restricted maximum likelihood (REML), and confidence intervals were calculated with the Hartung–Knapp adjustment. For studies providing paired data on ipsilateral and contralateral arteries, risk differences were calculated and combined using random-effects inverse-variance models. Between-study heterogeneity was quantified using I^2 and τ^2, with P values derived from Cochran's Q test. Sensitivity analyses were conducted to assess the influence of very low prevalence estimates. Specifically, the primary meta-analysis was repeated after excluding the study reporting the lowest prevalence. Leave-one-out analyses were also performed to evaluate the impact of individual studies on pooled estimates and heterogeneity.

Prespecified subgroup analyses were conducted according to stenosis site (intracranial vs extracranial) and ethnicity (Asian vs Caucasian populations). In addition, random-effects meta-analyses were performed to pool adjusted effect estimates for associations between dolichoectasia and individual cSVD markers, where comparable data were available.

All analyses were conducted in R (version 4.4.2; R Foundation for Statistical Computing, Vienna, Austria) using the meta package.

Supplemental Tables

Table S1. Distribution and severity of stenosis across intracranial and cervical arteries

Artery	Degree of stenosis, n (%)			
	< 50% or none	50-69%	70-99%	Occlusion
Intracranial artery (n=196)	156 (79.6%)	20 (10.2%)	16 (8.2%)	4 (2.0%)
Left ICA	192 (98%)	2 (1%)	1(0.5%)	1 (0.5%)
Right ICA	190 (96%)	2 (1%)	3 (1.5%)	1 (0.5%)
Left MCA	191 (97.4%)	3 (1.5%)	2 (1%)	0
Right MCA	187 (95.4%)	5 (2.6%)	4 (2.0%)	0
Left ACA	195 (99.5%)	0	0	1(0.5%)
Right ACA	195 (99.5%)	0	0	1(0.5%)
Left PCA	194 (99%)	0	2 (1%)	0
Right PCA	194 (99%)	0	1(0.5%)	1(0.5%)
BA	191 (97.5%)	4 (2.0%)	1(0.5%)	0
Left VA	193 (98.5%)	1(0.5%)	2 (1%)	0
Right VA	188 (95.9%)	6 (3.1%)	2 (1%)	0
Cervical ICA (n=220)				
Cervical part of left ICA	212 (96.4%)	4 (1.8%)	4 (1.8%)	0
Cervical part of right ICA	213 (96.8%)	3 (1.4%)	4 (1.8%)	0

Abbreviations: ICA, internal carotid artery; MCA, middle cerebral artery; ACA, anterior cerebral artery; PCA, posterior cerebral artery; BA, basilar artery; VA, vertebral artery.

Table S2. Distribution of intracranial artery diameters and basilar artery morphology

	Overall (N=229)	LAS (N=47)	No LAS (N=173)	*P* value	BADE(N=36)	No BADE(N=193)	*P* value
**Diameter, mm, mean (SD)**							
BA	3.27 (0.71)	3.25 (0.69)	3.28 (0.73)	.859	3.93 (0.63)	3.15 (0.66)	<.001
Left ICA	4.60 (0.70)	4.57 (0.79)	4.60 (0.67)	.762	4.83 (0.89)	4.56 (0.65)	.093
Right ICA	4.54 (0.78)	4.36 (1.02)	4.58 (0.70)	.487	4.79 (0.74)	4.49 (0.77)	.069
ICA (mean of left and right ICA)	4.57(0.66)	4.46 (0.75)	4.6 (0.63)	.287	4.81 (0.76)	4.53 (0.62)	.042
Left MCA	2.42 (0.37)	2.43 (0.37)	2.42 (0.36)	.852	2.56 (0.41)	2.39 (0.35)	.036
Right MCA	2.44 (0.41)	2.45 (0.48)	2.45 (0.39)	.355	2.72 (0.57)	2.39 (0.35)	<.001
MCA (mean of left and right MCA)	2.43 (0.33)	2.44 (0.36)	2.44 (0.33)	.965	2.64 (0.42)	2.39 (0.30)	<.001
**Height of BA bifurcation, n (%)**							
0	36 (15.7)	6 (12.8)	29 (16.8)	.485	0 (0)	36 (18.7)	<.001
1	166 (72.5)	38 (80.9)	120 (69.4)		9 (25)	157 (81.3)	
2	26 (11.4)	3 (6.4)	23 (13.3)		26 (72.2)	0	
3	1 (0.4)	0 (0)	1 (0.6)		1 (2.8)	0	
**Laterality score of BA, n (%)**							
0	115 (50.2)	24 (51.1)	86 (49.7)	.468	10 (27.8)	105 (54.4)	<.001
1	103 (45)	19 (40.4)	80 (46.2)		15 (41.7)	88 (45.6)	
2	10 (4.4)	4 (8.5)	6 (3.5)		10 (27.8)	0	
3	1 (0.4)	0 (0)	1 (0.6)		1 (2.8)	0	

Abbreviations: LAS, large artery stenosis; BADE, basilar artery dolichoectasia; SD, standard deviation; BA, basilar artery; ICA, internal carotid artery; MCA, middle cerebral artery.

Table S3. Anatomical distribution of 303 index infarcts in the per-infarct analysis

	Overall (N=303)	No LAS (N=209)	LAS (N=81)	No Embolic (N=163)	Embolic (N=140)	No BADE (N=260)	BADE (N=43)
**Small subcortical infarcts at index stroke (N=186)**							
Centrum semiovale	47 (15.5%)	38 (17.1%)	9 (11.1%)	31 (19%)	16 (11.4%)	39 (15%)	8 (18.6%)
Thalamus	36 (11.9%)	30 (13.5%)	6 (7.4%)	20 (12.3%)	16 (11.4%)	32 (12.3%0	4 (9.3%)
Internal capsule	32 (10.6%)	28 (12.6%)	4 (4.9%)	26 (16%)	6 (4.3%)	26 (10%)	6 (13.9%)
Pons	13 (4.3%)	10 (4.5%)	3 (3.7%)	8 (4.9%)	5 (3.6%)	8 (3.0%)	5 (11.6%)
Internal border zone	9 (3%)	2 (0.9%)	7 (8.6%)	2 (1.2%)	7 (5%)	9 (3.4%)	0
Lentiform nucleus	9 (3%)	9 (4.1%)	0 (0.0%)	7 (4.3%)	2 (1.4%)	7 (2.6%)	2 (4.6%)
Juxta-cortical	7 (2.3%)	5 (2.3%)	2 (2.5%)	3 (1.8%)	4 (2.9%)	6 (2.3%)	1 (2.3%)
Medulla	7 (2.3%)	6 (2.7%)	1 (1.2%)	4 (2.5%)	3 (2.1%)	5 (1.9%)	2 (4.6%)
Optic radiation	7 (2.3%)	3 (1.4%)	4 (4.9%)	3 (1.8%)	4 (2.9%)	5 (1.9%)	2 (4.6%)
Basal ganglia	6 (2%)	4 (1.8%)	2 (2.5%)	3 (1.8%)	3 (2.1%)	6 (2.3%)	0
Other	4 (1.3%)	3 (1.4%)	1 (1.2%)	2 (1.2%)	2 (1.4%)	4 (1.5%)	0
Splenium of corpus callosum	2 (0.7%)	1 (0.5%)	1 (1.2%)	1 (0.6%)	1 (0.7%)	0	2 (4.6%)
Cerebellum	2 (0.7%)	2 (0.9%)	0 (0.0%)	1 (0.6%)	1 (0.7%)	1 (0.3%)	1 (2.3%)
Midbrain	2 (0.7%)	2 (0.9%)	0 (0.0%)	2 (1.2%)	0 (0.0%)	2 (0.7%)	0
Anterior frontal	1 (0.3%)	1 (0.5%)	0 (0.0%)	1 (0.6%)	0 (0.0%)	0	1 (2.3%)
External capsule	1 (0.3%)	1 (0.5%)	0 (0.0%)	0 (0.0%)	1 (0.7%)	0	1 (2.3%)
Subcortical	1 (0.3%)	0 (0.0%)	1 (1.2%)	0 (0.0%)	1 (0.7%)	0	1 (2.3%)
**Cortical infarcts at index stroke (N=117)**							
Small cortical (cerebral hemisphere)	47 (15.5%)	33 (14.8%)	14 (17.3%)	17 (10.5%)	30 (21.4%)	43 (16.5%)	4 (9.3%)
Posterior borderzone	24 (7.9%)	17 (7.7%)	7 (8.6%)	15 (9.2%)	9 (6.4%)	23 (8.8%)	1 (2.3%)

< half PCA	19 (6.3%)	12 (5.4%)	7 (8.6%)	9 (5.5%)	10 (7.1%)	18 (6.9%)	1 (2.3%)
Small cortical (cerebellum)	6 (2%)	5 (2.3%)	1 (1.2%)	2 (1.2%)	4 (2.9%)	6 (2.3%)	0
<1/2 cerebellum hemisphere	6 (2%)	3 (1.4%)	3 (3.7%)	2 (1.2%)	4 (2.9%)	5 (1.9%)	1 (2.3%)
> half PCA	5 (1.7%)	2 (0.9%)	3 (3.7%)	1 (0.6%)	4 (2.9%)	5 (1.9%)	0
Anterior borderzone	3 (1%)	1 (0.5%)	2 (2.5%)	0 (0.0%)	3 (2.1%)	3 (1.1%)	0
Posterior half peripheral MCA	3 (1%)	1 (0.5%)	2 (2.5%)	1 (0.6%)	2 (1.4%)	3 (1.1%)	0
Anterior half peripheral MCA	2 (0.7%)	2 (0.9%)	0 (0.0%)	2 (1.2%)	0 (0.0%)	2 (0.7%)	0
< half ACA	1 (0.3%)	0 (0.0%)	1 (1.2%)	0 (0.0%)	1 (0.7%)	1 (0.3%)	0
>1/2 cerebellum hemisphere	1 (0.3%)	1 (0.5%)	0 (0.0%)	0 (0.0%)	1 (0.7%)	1 (0.3%)	0

Abbreviations: LAS, large artery stenosis; BADE, basilar artery dolichoectasia; MCA, middle cerebral artery; ACA, anterior cerebral artery; PCA, posterior cerebral artery.

Table S4. Distribution of posterior circulation index infarcts in the per-infarct analysis, stratified by presence or absence of BADE

	Overall (N=303)	No BADE (N=260)	BADE (N=43)
Total posterior circulation	130 (42.9%)	111 (42.7%)	19 (44.2%)
Thalamus	36 (11.9%)	32 (12.3%)	4 (9.3%)
Splenium of corpus callosum	2 (0.7%)	0	2 (4.6%)
Optic radiation	7 (2.3%)	5 (1.9%)	2 (4.6%)
Posterior borderzone	24 (7.9%)	23 (8.8%)	1 (2.3%)
< half PCA (cortical)	19 (6.3%)	18 (6.9%)	1 (2.3%)
> half PCA (cortical)	5 (1.7%)	5 (1.9%)	0
Small cortical in cerebellum	6 (2%)	6 (2.3%)	0
<1/2 cerebellum hemisphere	6 (2%)	5 (1.9%)	1 (2.3%)
>1/2 cerebellum hemisphere	1 (0.3%)	1 (0.3%)	0
Cerebellum white matter	2 (0.7%)	1 (0.3%)	1 (2.3%)
Brainstem	22 (7.3%)	15 (5.8%)	7 (16.3%)
Midbrain	2 (0.7%)	2 (0.7%)	0
Pons	13 (4.3%)	8 (3.0%)	5 (11.6%)
Medulla	7 (2.3%)	5 (1.9%)	2 (4.6%)

Abbreviations: BADE, basilar artery dolichoectasia; PCA, posterior cerebral artery.

Table S5. Anatomical distribution of 130 incident infarcts in the per-infarct analysis

Incident infarcts	Overall (N=130)	No LAS (N=95)	LAS (N=26)	No Embolic (N=73)	Embolic (N=57)	No BADE (N=80)	BADE (N=50)
Small subcortical infarcts on MRI (N=97)							
Centrum semiovale	32 (24.6%)	22 (21.2%)	10 (38.5%)	14 (19.2%)	18 (31.6%)	17 (21.2%)	15 (30%)
Optic radiation	13 (10%)	9 (8.7%)	4 (15.4%)	7 (9.6%)	6 (10.5%)	5 (6.2%)	8 (16%)
Juxta-cortical	13 (10%)	10 (9.6%)	3 (11.5%)	8 (11%)	5 (8.8%)	8 (10%)	5 (10%)
Thalamus	7 (5.4%)	7 (6.7%)	0 (0.0%)	7 (9.6%)	0 (0.0%)	3 (3.7%)	4 (8%)
Pons	7 (5.4%)	6 (5.8%)	1 (3.8%)	6 (8.2%)	1 (1.8%)	4 (5.0%)	3 (6%)
Internal border zone	6 (4.6%)	6 (5.8%)	0 (0.0%)	6 (8.2%)	0 (0.0%)	2 (2.5%)	4 (8%)
Lentiform nucleus	6 (4.6%)	6 (5.8%)	0 (0.0%)	5 (6.8%)	1 (1.8%)	5 (6.2%)	1 (2%)
Internal capsule	3 (2.3%)	1 (1%)	2 (7.7%)	1 (1.4%)	2 (3.5%)	3 (3.7%)	0
Anterior frontal	3 (2.3%)	2 (1.9%)	1 (3.8%)	2 (2.7%)	1 (1.8%)	2 (2.5%)	1 (2%)
Cerebellum	2 (1.5%)	2 (1.9%)	0 (0.0%)	2 (2.7%)	0 (0.0%)	1 (1.2%)	1 (2%)
Anterior temporal	1 (0.8%)	1 (1%)	0 (0.0%)	0 (0.0%)	1 (1.8%)	1 (1.2%)	0
External capsule	1 (0.8%)	1 (1%)	0 (0.0%)	1 (1.4%)	0 (0.0%)	1 (1.2%)	0
Splenium of corpus callosum	1 (0.8%)	1 (1%)	0 (0.0%)	0 (0.0%)	1 (1.8%)	1 (1.2%)	0
Subcortical	1 (0.8%)	1 (1%)	0 (0.0%)	1 (1.4%)	0 (0.0%)	1 (1.2%)	0
Other	1 (0.8%)	1 (1%)	0 (0.0%)	1 (1.4%)	0 (0.0%)	0	1 (2%)
Cortical infarcts on MRI (N=33)							
Small cortical (cerebral hemisphere)	9 (6.9%)	8 (7.6%)	1 (3.8%)	3 (4.1%)	6 (10.5%)	6 (7.4%)	3 (6%)
Posterior borderzone	6 (4.6%)	4 (3.8%)	2 (7.7%)	3 (4.1%)	3 (5.3%)	5 (6.2%)	1 (2%)
< half PCA	5 (3.8%)	4 (3.8%)	1 (3.8%)	1 (1.4%)	4 (7%)	4 (5%)	1 (2%)
Small cortical (cerebellum)	4 (3.1%)	3 (2.9%)	1 (3.8%)	1 (1.4%)	3(5.3%)	4 (5%)	0

Anterior borderzone	3 (2.3%)	3 (2.9%)	0 (0.0%)	1 (1.4%)	2 (3.5%)	3 (3.7%)	0
< half ACA	2 (1.5%)	2 (1.9%)	0 (0.0%)	0 (0.0%)	2 (3.5%)	1 (1.2%)	1 (2%)
Posterior half peripheral MCA	2 (1.5%)	2 (1.9%)	0 (0.0%)	1 (1.4%)	1 (1.8%)	2 (2.5%)	0
<1/2 cerebellum hemisphere	1 (0.8%)	1 (1%)	0 (0.0%)	1 (1.4%)	0 (0.0%)	0	1 (2%)
Anterior half peripheral MCA	1 (0.8%)	1 (1%)	0 (0.0%)	1 (1.4%)	0 (0.0%)	1 (1.2%)	0

Abbreviations: LAS, large artery stenosis; BADE, basilar artery dolichoectasia; MCA, middle cerebral artery; ACA, anterior cerebral artery; PCA, posterior cerebral artery.

Table S6. Sensitivity analyses: associations of large artery characteristics and embolic source with index stroke and incident infarcts, with additional adjustment for baseline cSVD score*

	LAS		BADE		ICAS		Any embolic source		Atrial fibrillation	
	Effect size (95% CI)	*P* value	Effect size (95% CI)	*P* value	Effect size (95% CI)	*P* value	Effect size (95% CI)	*P* value	Effect size (95% CI)	*P* value
**Index stroke**										
Subtype: lacunar v.s. non-lacunar	OR: 0.45 (0.21, 0.94)	0.035	OR: 4.45 (1.69, 13.1)	0.004	OR: 0.36 (0.15, 0.8)	0.014	OR: 0.48 (0.26, 0.87)	0.017	OR: 0.91 (0.34, 2.45)	0.858
Volume of index infarct, %ICV	β: 0.16 (-0.14, 0.45)	0.288	β: -0.05 (-0.39, 0.30)	0.791	β: 0.24 (-0.08, 0.56)	0.140	β: 0.08 (-0.16, 0.33)	0.503	β: 0.35 (-0.05, 0.74)	0.084
Number of index infarct	β: 0.53 (0.14, 0.93)	0.008	β: -0.16 (-0.60, 0.29)	0.484	β: 0.68 (0.29, 1.07)	<0.001	β: 0.25 (-0.07, 0.57)	0.121	β: 0.06 (-0.46, 0.59)	0.811
**Incident infarct**										
Presence of incident infarct	OR: 0.82 (0.33, 1.92)	0.656	OR: 1.61 (0.67, 3.83)	0.280	OR: 1.07 (0.40, 2.68)	0.885	OR: 1.42 (0.71, 2.84)	0.319	OR: 1.66 (0.55, 4.70)	0.352
Number of incident infarct	β: 0.14 (-0.26, 0.53)	0.494	β: 0.73 (0.29, 1.16)	0.001	β: 0.25 (-0.15, 0.65)	0.215	β: 0.21 (-0.11, 0.53)	0.195	β: 0.26 (-0.26, 0.78)	0.327
Symptomatic recurrence	OR: 2.54 (0.87, 7.18)	0.080	OR: 0.72 (0.18, 2.42)	0.623	OR: 3.57 (1.15, 10.9)	0.025	OR: 1.36 (0.52, 3.56)	0.528	β: 1.58 (0.32, 5.85)	0.527

*Models were adjusted for age, sex, vascular risk factors (smoking, hypertension, diabetes mellitus, hyperlipidemia, and body mass index), and baseline summary cSVD score. The OR was estimated using binary logistic regression, and the β using linear regression.

Abbreviations: LAS, large artery stenosis; BADE, basilar artery dolichoectasia; ICAS, intracranial atherosclerotic stenosis; OR, odds ratio; CI, confidence interval; ICV, intracranial volume; TIA, transient ischemic attack

Table S7. Sensitivity analysis: associations between ICAS, embolic source, arterial diameters and cSVD markers*

	ICAS		Any embolic source		Atrial fibrillation		ICA diameter†		MCA diameter†		BA diameter†	
	Effect size (95% CI)	*P* value	Effect size (95% CI)	*P* value	Effect size (95% CI)	*P* value	Effect size (95% CI)	*P* value	Effect size (95% CI)	*P* value	Effect size (95% CI)	*P* value
Summary cSVD score	OR: 0.97 (0.50, 1.87)	0.919	OR: 0.82 (0.50, 1.34)	0.427	OR: 0.54 (0.25, 1.17)	0.119	OR: 2.00 (1.35, 2.98)	<0.001	OR: 3.46 (1.58, 7.73)	0.002	OR: 1·71 (1·18, 2·51)	0.006
Number of lacunes	β: 0.71 (-0.36, 1.78)	0.192	β: 0.13 (-0.67, 0.93)	0.749	β: -0.18 (-1.48, 1.12)	0.786	β: 1.02 (0.41, 1.63)	0.001	β: 1.79 (0.58, 3.00)	0.004	β: 0·88 (0·31, 1·46)	0.003
Number of microbleeds	β: 0.84 (-1.02, 2.70)	0.373	β: 0.05 (-1.25, 1.36)	0.937	β: -0.40 (-2.52, 1.72)	0.709	β: 1.27 (0.25, 2.29)	0.015	β: 2.17 (0.16, 4.18)	0.035	β: 0·29 (-0·68, 1·26)	0.560
WMH volume, %ICV	β: -0.02 (-0.17, 0.12)	0.745	β: -0.02 (-0.13, 0.09)	0.711	β: -0.06 (-0.24, 0.12)	0.504	β: 0.05 (-0.04, 0.14)	0.286	β: 0.30 (0.14, 0.47)	<0.001	β: 0·07 (-0·01, 0·16)	0.078
BG PVS volume, %ROIV	β: -0.06 (-0.12, 0.01)	0.088	β: -0.07 (-0.12, -0.03)	0.002	β: -0.03 (-0.11, 0.04)	0.388	β: 0.05 (0.01, 0.08)	0.013	β: 0.05 (-0.02, 0.13)	0.158	β: 0·04 (0·00, 0·07)	0.028
CSO PVS volume, %ROIV	β: -0.03 (-0.12, 0.07)	0.589	β: -0.06 (-0.13, 0.01)	0.069	β: -0.03 (-0.14, 0.09)	0.624	β: -0.01 (-0.06, 0.05)	0.79	β: 0.02 (-0.09, 0.13)	0.702	β: 0·02 (-0·03, 0·08)	0.348
Total PVS volume, %ROIV	β: -0.03 (-0.11, 0.05)	0.482	β: -0.07 (-0.13, -0.01)	0.031	β: -0.03 (-0.13, 0.07)	0.533	β: 0.00 (-0.04, 0.05)	0.866	β: 0.03 (-0.07, 0.12)	0.556	β: 0·03 (-0·02, 0·07)	0.207
Brain volume, %ICV	β: -0.33 (-1.45, 0.79)	0.565	β: -0.24 (-1.11, 0.64)	0.596	β: -0.54 (-1.97, 0.88)	0.453	β: 0.99 (0.30, 1.67)	0.005	β: -0.30 (-1.67, 1.06)	0.662	β: -0.21 (-0.86, 0.44)	0.521
WMH progression, %ICV	β: 0.00 (-0.13, 0.13)	0.983	β: 0.06 (-0.04, 0.16)	0.239	β: 0.11 (-0.08, 0.29)	0.251	β: -0.07 (-0.15, 0.01)	0.102	β: 0.17 (0.01, 0.33)	0.036	β: -0·01 (-0·09, 0·06)	0.770

* Models were adjusted for age, sex, vascular risk factors (smoking, hypertension, diabetes mellitus, hyperlipidemia, and body mass index. For brain volume, models were additionally adjusted for the number of lacunes. For WMH progression, models were additionally for baseline cSVD score. β coefficients were estimated using linear regression, and OR using ordinal logistic regression.

† For models including continuous arterial diameters as exposures, LAS status was additionally included as a covariate.

Abbreviations: ICAS, intracranial artery stenosis; cSVD, cerebral small vessel disease; OR, odds ratio; CI, confidence interval; WMH, white matter hyperintensity; ICV, intracranial volume; ROIV, region of interest volume; BG PVS, basal ganglia perivascular space; CSO PVS, centrum semiovale perivascular space; ICA, internal carotid artery; MCA, middle cerebral artery; BA, basilar artery.

Table S8. Sensitivity analysis: models including LAS×BADE interaction for index stroke, incident infarcts, and cSVD markers

	LAS		BADE		Interaction (LAS×BADE)	
	Effect size (95% CI)	*P* value	Effect size (95% CI)	*P* value	Effect size (95% CI)	*P* value
**Index stroke***						
Subtype: lacunar v.s. non-lacunar	OR: 0.50 (0.22, 1.12)	0.097	OR: 4.31 (1.42, 15.39)	0.015	OR: 0.59 (0.06, 7.01)	0.657
Volume of index infarct, %ICV	β: 0.19 (-0.12, 0.51)	0.230	β: 0.06 (-0.33, 0.45)	0.770	β: -0.27 (-1.16, 0.61)	0.545
Number of index infarct	β: 0.52 (0.10, 0.95)	0.016	β: -0.14 (-0.64, 0.35)	0.565	β: 0.04 (-1.09, 1.17)	0.944
**Incident infarct***						
Presence of incident infarct	OR: 1.01 (0.37, 2.54)	0.991	OR: 2.03 (0.76, 5.37)	0.154	OR: 0.36 (0.03, 3.40)	0.376
Number of incident infarct	β: 0.15 (-0.27, 0.56)	0.486	β: 0.75 (0.26, 1.23)	0.003	β: 0.21 (-0.89, 1.32)	0.708
Symptomatic recurrence	β: 2.70 (0.85, 8.35)	0.084	β: 0.77 (0.14, 3.23)	0.734	β: 0.60 (0.02, 9.19)	0.728
**cSVD markers†**						
Summary cSVD score	OR: 1.05 (0.55, 2.00)	0.881	OR: 2.40 (1.09, 5.36)	0.03	OR: 1.43 (0.27, 7.74)	0.676
Number of lacunes	β: 0.52 (-0.50, 1.54)	0.315	β: 1.95 (0.78, 3.12)	0.001	β: 2.44 (-0.27, 5.15)	0.078
Number of microbleeds	β: 1.05 (-0.65, 2.76)	0.225	β: 3.55 (1.58, 5.52)	<0.001	β: -0.13 (-4.67, 4.41)	0.955
WMH volume, %ICV	β: -0.02 (-0.17, 0.12)	0.763	β: 0.25 (0.08, 0.42)	0.004	β: 0.04 (-0.35, 0.43)	0.825
BG PVS volume, %ROIV	β: -0.04 (-0.10, 0.02)	0.226	β: 0.11 (0.03, 0.18)	0.004	β: -0.03 (-0.20, 0.13)	0.712
CSO PVS volume, %ROIV	β: 0.00 (-0.09, 0.09)	0.954	β: 0.14 (0.04, 0.25)	0.009	β: -0.16 (-0.41, 0.08)	0.178
Total PVS volume, %ROIV	β: -0.00 (-0.08, 0.08)	0.908	β: 0.13 (0.04, 0.22)	0.005	β: -0.13 (-0.34, 0.08)	0.229
Brain volume, %ICV	β: -0.45 (-1.61, 0.70)	0.442	β: -0.36 (-1.72, 1.00)	0.600	β: 2.80 (-0.29, 5.89)	0.076
WMH progression, %ICV	β: -0.02 (-0.16, 0.11)	0.727	β: 0.16 (0.00, 0.31)	0.047	β: -0.07 (-0.42, 0.28)	0.688

*Models were adjusted for age, sex, vascular risk factors (smoking, hypertension, diabetes mellitus, hyperlipidemia, and body mass index), and baseline summary cSVD score. †Models were adjusted for age, sex, and vascular risk factors. For brain volume, models were additionally adjusted for the number of lacunes. For WMH progression, models were additionally for baseline cSVD score. β coefficients were estimated using linear regression, and OR using logistic regression.

Abbreviations: LAS, large artery stenosis; BADE, basilar artery dolichoectasia; cSVD, cerebral small vessel disease; OR, odds ratio; CI, confidence interval; WMH, white matter hyperintensity; ICV, intracranial volume; ROIV, region of interest volume; BG PVS, basal ganglia perivascular space; CSO PVS, centrum semiovale perivascular space;

Table S9. Comparison of the ipsilateral and contralateral large artery stenosis in lacunar stroke

Study	Year	Ethnicity	Study population	LS No.	Laterality of stenosis, n		Severity of stenosis	Stenosis evaluation modality	Stenosis location	Definition of lacunar stroke
					Ipsi-lateral*	Contral-lateral†				
Norrving[45]	1989	European	Consecutive hospital patients	61	2	2	>50%	Catheter	Extra+Intra	Clinical+Imaging
Boiten[46]	1991	European	Not mentioned if consecutive	86	11	6	>50%	NA	Extra	Clinical+Imaging
Tegeler[47]	1991	US	Patients referred to a neurosonography lab	55	7	2	>50%	DUS	Extra	Clinical+Imaging
Landi[48]	1992	European	Consecutive hospital patients	88	16	-	>50%	DUS	Extra	Clinical
Boiten[49]	1996	European	ECST	222	43	17	>70%	Catheter	Extra	Imaging
Mead[50]	1998	European	Consecutive hospital patients	80	9	9	>50%	DUS	Extra	Clinical
Besson[51]	2000	European	Consecutive hospital patients	200	2	12	>50%	DUS	Extra	Clinical+Imaging
Mead[10]	2002	European	Consecutive hospital patients	259	14	10	>80%	DUS+TCD	Extra	Clinical+Imaging
Baumgartner[52]	2003	European	Consecutive hospital patients	244	53	-	>50%	DUS+TCD	Extra/Intra	Clinical+Imaging
Mok[53]	2003	Asian	Consecutive hospital patients	71	15	-	>50%	DUS+MRA/CTA	Extra+Intra	Imaging
Tejada[11]	2003	European	Consecutive hospital patients	135	30	12	>50%	DUS	Extra	Clinical+Imaging
Wessels[54]	2005	European	Consecutive hospital patients	73	9	-	>50%	DUS+TCD	Extra+Intra	Clinical
Kim[55]	2008	Asian	Consecutive hospital patients	167	36	-	>50%	MRA	Intra	Imaging
Mecheli[56]	2008	European	Consecutive hospital patients	206	24	-	>50%	DUS+MRA/CTA	Extra+Intra	Clinical+Imaging
Jackson[9]	2010	European	Hospital based and community-based patients	1062	48	32	>70%	DUS	Extra	Clinical+Imaging
Kim[57]	2010	Asian	Consecutive hospital patients	86	19	15	>50%	MRA	Intra	Imaging

Wardlaw[58]	2011	European	Consecutive hospital patients	67	6	-	>50%	TCD	Extra+Intra	Clinical+Imaging
Asdaghi[28]	2014	Global	SPS3	1679	26	-	>50%	MRA/CTA	Intra	Clinical+Imaging
Van[59]	2014	European	Consecutive hospital patients	343	43	-	>50%	CTA	Extra+Intra	Clinical
Duan[60]	2015	Asian	Consecutive hospital patients	312	100	169	>50%	MRA	Extra+Intra	Imaging
Muscari[61]	2016	European	Not mentioned if consecutive	55	7	-	>50%	DUS	Extra	Clinical+Imaging
Suzuki[62]	2016	Asian	Consecutive hospital patients	64	8	-	>50%	DUS+MRA/CTA	Extra+Intra	Imaging
Eppinger[63]	2019	European	Consecutive hospital patients	335	30	33	>50%	DUS+TCD	Extra+Intra	Imaging
Naess[64]	2019	European	Consecutive hospital patients	294	16	-	>50%	MRA/CTA	Extra+Intra	Clinical+Imaging
Liu[65]	2023	Asian	CHANCE-2	2143	340	-	>50%	MRA/CTA	Intra	Imaging
Wei[66]	2024	Asian	CATIS-2	997	116	-	>50%	MRA	Intra	Imaging
Current study	2026	European	Consecutive hospital patients	131	7	12	>50%	DUS+MRA/CTA	Extra+Intra	Clinical+Imaging

* Ipsilateral, or upstream, or relevant, or symptomatic stenosis of the lacunar infarcts

† Contralateral, or non-upstream, or non-relevant, or asymptomatic stenosis of the lacunar infarcts

Abbreviations: CATIS-2, China Antihypertensive Trial in Acute Ischemic Stroke II; CHANCE, Clopidogrel in High-risk patients with Acute Non-disabling Cerebrovascular Events Trial; CTA, CT angiography; DUS, doppler ultrasonography; ECST, European Carotid Surgery Trial; Extra, extracranial artery; Intra, intracranial artery; LS, lacunar stroke; MRA, MR angiography; SPS3, The Secondary Prevention of Small Subcortical Strokes Trial; TCD, transcranial doppler.

Table S10. Studies evaluating the association between large artery stenosis and SBI/lacunes

Hospital based study										
Author, Year	N	Study population	Mean Age	Evaluation of stenosis			SBI		Findings	Association
				Location	Imaging	Severity	Size, mm	%		
Boon, 1994[67]	755	Patients with first-ever supratentorial IS	71	Extra	DUS	>50%	NA	27	Carotid stenosis was not significantly associated with any type of SBI (OR 0.61, *P*=0.08).	ns.
Uehara, 1999[68]	219	Subjects underwent check-ups	63	Extra<br>Intra	MRA	25/50/75%	5-15	40.2	BG-SBI: related to carotid stenosis but not ICAS; WM-SBI: not related to carotid stenosis and ICAS.	BG: positive<br>WM: ns.
Geerlings, 2010[69]	1044	Subjects with atherosclerosis	58	Extra	DUS	>50%	>3	26	SBI was significantly associated with carotid artery stenosis (OR 2.36).	Positive
Li, 2016[70]	455	Patients with TIA	66	Extra<br>Intra	DUS, MRA, CTA	>50%	3-15	66	SBI was associated with extracranial carotid stenosis (OR 1.88, *P*=0.02), but not with ICAS (OR 1.47, *P*=0.15).	Extra: positive<br>Intra: ns.
Kigka, 2024[71]	196	Patients with asymptomatic carotid stenosis	70	Extra	DUS	>50%	3-15	16.9	Carotid stenosis severity did not differ significantly between subjects with and without SBIs (p = 0.767).	ns.
Lin, 2024[72]	239	Patients with RSSI	60	Intra	VWI	>50%	3-15	41.3	Severity of MCA stenosis was not associated with lacunes (*P*=0.178).	ns.
Current study	229	Patients with mild stroke	66	Extra<br>Intra	DUS, MRA, CTA	>50%	3-15	52.4	Large artery stenosis was not associated with numbers of lacunes (*P*=0.139).	ns.

Community based study										
Author, Year	N	Study population	Mean Age	Evaluation of stenosis			SBI		Findings	Association
				Location	Imaging	Severity	Size	%		
Manolio, 1999[73]	3502	Cardiovascular Health study	75	Extra	DUS	25/50/75%	>3	30.5	SBI was significantly associated with severity or carotid stenosis (*P*=0.002).	Positive
Takahashi, 2005[74]	747	Subjects undergoing health screening	63	Extra Intra	DUS, MRA	25/50%	3-20	19	SBI was significantly associated with large artery stenosis.	Positive
Das, 2008[75]	2040	Framingham Offspring study	62	Extra	DUS	>25%	>3	10.7	Carotid stenosis was associated with SBIs (OR 1.62, *P*=0.009).	Positive
Zhai, 2018[76]	1237	Community based study in rural China	57	Intra	MRA	Any degree	3-15	17.9	ICAS was significantly associated with lacunes (OR 2.91, *P*<0.001).	Positive
Wang, 2023[77]	3061	Community based study in China	61	Intra	VWI	>50%	3-15	5.6	ICAS was significantly associated with lacunes (OR 2.63, *P*<0.001).	Positive
Ackah, 2025[78]	225	Community based study in China	65	Intra	VWI	Any degree	3-15	12.9	Severity of ICAS was associated with lacunes (OR 2.17, *P*=0.032).	Positive

Abbreviations: BG, basal ganglia; DUS, doppler ultrasonography; Extra, extracranial artery; ICAS, intracranial artery stenosis; Intra, intracranial artery; IS, ischemic stroke; MRA, MR angiography; ns., non-significant; OR, odds ratio; RSSI, recent small subcortical infarcts; SBI, silent brain infarct; TIA, transient ischemic attack; VWI, vessel wall imaging; WM, white matter.

Table S11. Studies evaluating the laterality of SBI/lacunes by the side of large artery stenosis

Author, Year	N	Study population	Mean Age	SBI size/type	SBI laterality relative to stenosis (%)		Findings	Association
					Ipsi-	Contra-		
Brott, 1994[79]	848	Patients with carotid stenosis>60%	NA	NA	43	44	SBIs showed no lateral predominance relative to the stenosed artery.	ns.
Manolio, 1999[73]	178	Cardiovascular Health study	75	>3 mm	31.2	29.3	No stronger association of SBI was found with ipsilateral versus contralateral stenosis.	ns.
Uehara, 1999[68]	219	Subjects underwent vascular check-ups	63	5-15mm	39.4	33.3	Prevalence of SBI at basal ganglia did not differ significantly by side of carotid stenosis.	ns.
Baradaran, 2016[80]	104	Patients with unilateral carotid stenosis >50%	NA	Lacunar	19.6	13.7	Carotid stenosis was associated with more downstream cortical, but not lacunar, SBIs.	Cortical: positive<br>Lacunar: ns.
				Cortical	14.7	3.9		
Benli, 2021[81]	69	Patients with unilateral carotid stenosis >50%	73	All	17.4	5.8	SBI number, location, and size did not differ by side of carotid stenosis.	ns.
				3-15mm	7.2	2.9		
				>15mm	10.1	2.9		
Wang, 2022[82]	257	Patients with unilateral carotid stenosis >50%	70	>3 mm	48.6	35.8	Prevalence of ipsilateral SBI was higher than contralateral (OR 1.70, P=0.005).	Positive
Lin, 2023[83]	183	Patients with >50% carotid stenosis	71	>3 mm	14.9	6.1	Prevalence of SBI was greater on the side of >70% carotid stenosis.	Positive
Wu, 2023[84]	219	Patients with unilateral MCA occlusion	57	3-15mm	43.4	25.1	Uni-MCA occlusion has a higher prevalence of lacune in the ipsilateral hemisphere.	Positive
Zheng, 2024[85]	112	Patients with ICAS	63	3-15mm	35.7	26.8	Lacune presence did not differ by side (P=0.1), but counts were higher ipsilateral to ICAS.	ns.

Abbreviations: ICAS, intracranial artery stenosis; MCA, middle cerebral artery; SBI, silent brain infarct.

Table S12. Studies evaluating the association between large artery stenosis and white matter hyperintensities

Hospital based study									
Author, Year	N	Study population	Mean Age	Evaluation of stenosis			WMH rating method	Findings	Association
				Location	Imaging	Severity			
Pu, 2009[86]	185	Patients with acute IS	56	Extra Intra	MRA	>50%	Visual rating (ARWMC[87])	Cerebral large artery stenosis was not related to WMH (OR 0.97, P=0.94).	ns.
Lee, 2011[88]	268	Patients with acute IS or TIA	67	Extra Intra	MRA	>50%	Visual rating (Scheltens[89])	WMH was associated with intracranial (P=0.001) rather than extracranial artery stenosis (P=0.21).	Intra: positive. Extra: ns.
Schulz, 2013[90]	671	Patients with TIA or minor stroke	70	Extra	DUS, CTA, MRA	>50%	Visual rating (ARWMC)	The presence, severity of, and risk factors for carotid stenosis were not associated with WMHs.	ns.
Park, 2015[91]	697	Patients with acute IS	68	Extra Intra	MRA	>50%	Visual rating (Fazekas[92])	ICAS was associated with higher DWMH and PVH scores (both P<0.001).	Positive
Duan, 2018[93]	2420	Patients with acute IS or TIA	62	Extra Intra	MRA	>50%	Visual rating (Manolio[73])	WMH was associated with ICAS (OR 1.39, P=0.002).	Positive
Wang, 2022[94]	516	Patients with ICAS	59	Intra	MRA	>70%	Visual rating (Fazekas)	ICAS >70% was not associated WMH (OR 1.03, P=0.89).	ns.
Lin, 2024[72]	239	Patients with RSSI	60	Intra	VWI	>50%	Visual rating (Fazekas)	Severity of MCA stenosis was not associated with DWMH (P=0.26) and PVH (P=0.384).	ns.
Current study	229	Patients with mild stroke	66	Extra Intra	DUS, MRA, CTA	>50%	Visual rating (Fazekas) and Volumetric	Large artery stenosis was not associated with WMH volume (P=0.44).	ns.

Community based study									
Author, Year	N	Study population	Mean Age	Evaluation of stenosis			WMH rating method	Findings	Association
				Location	Imaging	Severity			
Manolio, 1999[73]	3502	Cardiovascular Health study	75	Extra	DUS	25/50/75 %	Visual rating (Manolio)	WMH was not associated with increasing severity of stenosis (*P*=0.19)	ns.
Romero, 2016[95]	1971	Framingham Offspring study	58	Extra	DUS	>50%	Volumetric	WMH volume was related to carotid stenosis (OR 2.35, *P*=0.03)	Positive
Zhai, 2018[76]	1237	Community based study in rural China	57	Intra	MRA	Any degree	Volumetric	ICAS was associated WMH volume (β 0.54±0.13, *P*<0.001)	Positive
Del, 2020[96]	581	Community based study in rural Ecuador	71	Intra	MRA	>50%	Visual rating (Pantoni[97])	WMH was associated with ICAS (OR 3.67, *P*=0.003)	Positive
Choi, 2022[98]	1337	Participants underwent check-ups	52	Intra	MRA	>50%	Visual rating (Fazekas)	ICAS was associated with DWMH (OR 7.11, *P*=0.001) and PVH (OR 3.97, *P*=0.015)	Positive
Ackah, 2025[78]	225	Community based study in China	65	Intra	VWI	Any degree	Visual rating (Manolio)	Severity of ICAS was associated with WMH burden (OR 2.45, *P*=0.015)	Positive

Abbreviations: ARWMC, age-related white matter changes; CTA, CT angiography; DUS, doppler ultrasonography; DWMH, deep white matter hyperintensity; Extra, extracranial artery; ICAS, intracranial artery stenosis; Intra, intracranial artery; IS, ischemic stroke; MRA, MR angiography; ns., non-significant; OR, odds ratio; PVH, periventricular hyperintensity; RSSI, recent small subcortical infarct. TIA, transient ischemic attack; WMH, white matter hyperintensity.

Table S13. Studies evaluating the lateral association between large artery stenosis and white matter hyperintensities

Author, Year	N	Study population	Mean Age	Evaluation of stenosis			WMH rating method	Findings	Association
				Location	Imaging	Severity			
Streifler, 1995[99]	1197	Patients enrolled in NASCET	65	Extra	DUS	30/70%	Visual rating	WMH not related to the degree of ipsilateral stenosis (severe vs. mild OR 1.08, *P*=0.95).	ns.
Altaf, 2008[100]	190	Patients with symptomatic carotid artery disease	70	Extra	Black blood MRI	30/50/70%	Volumetric	WMH volume not related to the degree of ipsilateral stenosis (*P*=0.60).	ns.
Saba, 2009[101]	147	Consecutively registered patients undergoing MDCT	74	Extra	MDCT	40/70%	Visual rating (ARWMC)	There was a significant association between carotid stenosis and ipsilateral WMH (OR 1.37, *P*=0.01).	Positive
Potter, 2012[102]	500	Patients with stroke or TIA	68	Extra	DUS	50/70%	Visual rating (Fazekas)	There was no association between carotid stenosis and ipsilateral or contralateral WMH.	ns.
Fang, 2020[103]	180	Patients with IS or TIA and a 50-99% MCA stenosis	64	Intra	MRA	>50%	Visual rating (ARWMC)	The severity of MCA stenosis was not associated with ipsilateral WMH (*P*=0.93).	ns.
Benli, 2021[81]	69	Patients with unilateral carotid stenosis >50%	73	Extra	DUS	>50%	Visual rating (Fazekas)	There was no hemispheric difference in DWMH and PVH.	ns.
Huang, 2022[104]	161	Patients with unilateral carotid stenosis	66	Extra Intra	DSA, MRA, DUS	>50%	Visual rating (Fazekas)	PVH and DWMH correlated more with ipsilateral than contralateral stenosis severity.	Positive

Wang, 2022[82]	257	Patients with unilateral carotid stenosis >50%	70	Extra Intra	DUS, MRA	>50%	Visual rating (Fazekas)	No significant hemispheric asymmetry in PVH or DWMH relative to ICA stenosis.	ns.
Lin, 2023[83]	183	Patients with >50% carotid stenosis	71	Extra	DUS	>50%	Volumetric	Patients had greater WMH volume ipsilateral to the side of stenosis than the contralateral side (*P*=0.046).	Positive
Wu, 2023[84]	219	Patients with unilateral MCA occlusion	57	Intra	CTA, DSA	Occlusion	Visual rating (ARWMC)	WMH showed no interhemispheric difference in patients with unilateral MCA occlusion.	ns.
Feng, 2023[105]	150	Patients with unilateral anterior-circulation ICAS	63	Extra Intra	CTA	>50%	Visual rating (Fazekas)	There was no significant association between severity of stenosis and DWMH and PVH.	ns.
Zheng, 2024[85]	112	Patients with anterior-circulation ICAS	63	Intra	CTA	>50%	Visual rating (Fazekas)	PVH and DWMH were more severe in ipsilateral than contralateral hemispheres.	Positive

Abbreviations: ARWMC, age-related white matter changes; CTA, CT angiography; DUS, doppler ultrasonography; DWMH, deep white matter hyperintensity; DSA, digital subtraction angiography; Extra, extracranial artery; Intra, intracranial artery; IS, ischemic stroke; MCA, middle cerebral artery; MDCT, multidetector computed tomography; MRA, MR angiography; NASCET, North American Symptomatic Carotid Endarterectomy Trial; ns., non-significant; OR, odds ratio; PVH, periventricular hyperintensity; TIA, transient ischemic attack; WMH, white matter hyperintensity.

Table S14. Studies evaluating the association between large artery stenosis and cerebral microbleeds

Author, Year	N	Study population	Mean Age	Evaluation of stenosis			CMB		Findings	Association
**Hospital based study**										
				Location	Imaging	Severity	MRI	%		
Chung, 2014[106]	834	Patients with acute IS or TIA	67	Extra Intra	CTA	>50%	3.0T, SWI	40.2	Severe ICA calcification related to CMBs (OR 2.86, *P*<0.001).	Positive
Peng, 2014[107]	90	Patients with acute IS	67	Extra Intra	TCD, DUS	>50%	1.5T, GRE	33.3	There were no significant relationships between CMBs and large artery stenosis.	ns.
Song, 2015[108]	220	Patients with acute IS	64	Extra Intra	MRA, CTA, DSA	>50%	1.5T, GRE	20.9	Cerebral artery stenosis was not significantly associated with CMBs.	ns.
Boulouis, 2016[109]	343	Patients with symptomatic ICH	71	Intra	CTA	>50%	1.5T, GRE	46	Intracranial stenosis was not associated with CMBs (OR 0.99, *P*=0.86).	ns.
Wang, 2022[94]	516	Patients with intracranial LAD	59	Intra	MRA	>70%	3.0T, SWI	38.6	The severity of LAD was not associated with CMBs (OR 1.21, *P*=0.45).	ns.
Lin, 2024[72]	239	Patients with RSSI	60	Intra	VWI	>50%	3.0T, SWI	48.1	Severity of MCA stenosis was not associated with CMBs (*P*=0.228).	ns.
Current study	229	Patients with mild stroke	66	Extra Intra	DUS, MRA, CTA	>50%	3.0T, SWI	18.7	Large artery stenosis was not associated with the number of CMBs (*P*=0.289)	ns.
**Community based study**										
**Author, Year**	**N**	**Study population**	**Mean Age**	**Evaluation of stenosis**			**CMB**		**Findings**	**Association**
				Location	Imaging	Severity	MRI	%		
Romero, 2016[95]	1243	Framingham offspring study	57	Extra	DUS	25/50%	1.5T, GRE	1.6	CMB was mot associated with >50% carotid stenosis (OR 1.99, *P*=0.35).	Positive

Zhai, 2018[76]	1237	Community based study in rural China	57	Intra	MRA	Any degree	3.0T, SWI	11.3	CMB was not associated with ICAS (OR 1.28, *P*=0.30).	ns.
Del, 2020[96]	581	Community based study in rural Ecuador	71	Intra	MRA	>50%	1.5T, GRE	11	CMB was not associated with ICAS (OR 0.48, *P*=0.47).	ns.
Wang, 2023[77]	3061	Community based study in China	61	Intra	VWI	>50%	3.0T, SWI	10.2	ICAS was not associated with presence (OR 1.31, *P*=0.29) or burden (OR 1.35, *P*=0.23) of CMBs.	ns.
Ackah, 2025[78]	225	Community based study in China	65	Intra	VWI	Any degree	3.0T, SWI	14.7	Severity of ICAS was not associated with CMBs (*P*=0.943).	ns.

Abbreviations: CMB, cerebral microbleeds; CTA, CT angiography; DUS, doppler ultrasonography; Extra, extracranial artery; GRE, gradient recall echo; ICAS, intracranial artery stenosis; ICH, intracranial hemorrhage; Intra, intracranial artery; IS, ischemic stroke; LAD, large artery disease; MRA, MR angiography; ns., non-significant; OR, odds ratio; TCD, transcranial doppler; SWI, Susceptibility-Weighted Imaging; TIA, transient ischemic attack; VMI, vessel wall imaging.

Table S15. Studies evaluating the association between large artery stenosis and perivascular spaces

Author, Year	N	Study population	Mean Age	Evaluation of stenosis			PVS rating method	Findings	Association
**Hospital based study**									
				Location	Imaging	Severity			
Shen, 2020[110]	202	Patients underwent cerebrovascular DSA	62	Extra Intra ICA	DSA	>70%	Zhu[111]	CS-PVS but not BG-PVS was associated with ICA stenosis.	CS-PVS: positive. BG-PVS: ns.
Du, 2020[112]	185	Patients with MCA stenosis	63	Intra	MRA	>50%	Doubal[113]	MCA stenosis was associated with BG-PVS (OR 3.67, *P*=0.023).	BG-PVS: positive
Wang, 2022[94]	516	Patients with ICAS	59	Intra	MRA	>70%	Doubal	CS-PVS but not BG-PVS was associated with the severity of ICAS.	CS-PVS: positive. BG-PVS: ns.
Han, 2023[114]	177	Patients of moyamoya disease/syndrome	42	Intra	VWI	>70%	Zhu	Severe MCA stenosis was associated with CS-PVS burden (OR=6.26, *P*<0.001).	CS-PVS: ns.
Lin, 2024[72]	239	Patients with RSSI	60	Intra	VWI	>50%	Doubal	Severe MCA stenosis was not associated with CS-PVS (*P*=0.271) and BG-PVS (*P*=0.435).	CS-PVS: ns. BG-PVS: ns.
Current study	229	Patients with mild stroke	66	Extra Intra	DUS, MRA, CTA	>50%	Doubal	Large artery stenosis was not associated with CS-PVS (*P*=0.178) and BG-PVS (*P*=0.206).	CS-PVS: ns. BG-PVS: ns.
**Community based study**									
**Author, Year**	**Patient No.**	**Subjects**	**Mean Age**	**Evaluation of stenosis**			**PVS rating method**	**Findings**	**Association**
				**Location**	**Imaging**	**Severity**			

Zhai, 2018[76]	1237	Community based study in rural China	57	Intra	MRA	Any degree	Zhu	ICAS was not associated with BG-PVS (OR 1.0, *P*=0.99) and CS-PVS (OR 1.04, *P*=0.81).	CS-PVS: ns. BG-PVS: ns.
Del, 2020[96]	581	Community based study in rural Ecuador	71	Intra	MRA	>50%	Doubal	ICAS was not associated with BG-PVS (OR 0.98, *P*=0.96).	BG-PVS: ns.
Wang, 2023[77]	3061	Community based study in China	61	Intra	VWI	>50%	Doubal	ICAS >50% was not associated with severity of BG-PVS.	BG-PVS: ns.
Ackah, 2025[78]	225	Community based study in China	65	Intra	VWI	Any degree	Doubal	Severity of ICAS was associated with PVS burden (OR 3.11, *P*<0.001).	Positive

Abbreviations: BG-PVS, basal ganglia perivascular spaces; CS-PVS, centrum semiovale-perivascular spaces; CTA, CT angiography; DSA, digital subtraction angiography; DUS, doppler ultrasonography; Extra, extracranial; ICA, internal carotid artery; ICAS, intracranial artery stenosis; Intra, intracranial; IS, ischemic stroke; MCA, middle cerebral artery; MRA, MR angiography; ns., non-significant; OR, odds ratio; PVS, perivascular spaces; VMI, vessel wall imaging.

Table S16. Studies evaluating the association between intracranial artery dolichoectasia and cSVD

Author, Year	N	Study population	cSVD markers investigated							Dilation†	Findings
			LS	Lacune	WMH	CMB	PVS	Atrophy	Incident infarcts*		
Ince, 1998[115]	387	Stroke patients	√	×	×	×	×	×	×	Anterior Posterior	Patients with IADE were more likely to present with a lacunar infarction subtype compared with those without IADE (42% vs. 17%, *P*=0.04).
Pico, 2003[116]	510	Stroke patients	√	×	×	×	×	×	×	Anterior Posterior	IADE was associated with a higher odds of lacunar infarction compared with atherothrombotic infarction (OR 2.89, *P*=0.01).
Pico, 2005[117]	510	Stroke patients	√	√	√	×	√	×	×	Anterior Posterior	IADE was associated with multi-lacuna (OR 2.05, *P*=0.027), WMH (OR 2.40, *P*=0.026), and PVS (OR 3.6, *P*=0.007).
Pico, 2007[42]	381	Autopsy stroke patients	√	√	×	×	√	×	×	Anterior Posterior	IADE was associated with small vessel pathology (OR 3.85, *P*=0.004), lacunar infarction (OR 2.42, *P*=0.03), and multi-lacuna (OR 2.85, *P*=0.04), but not with PVS.
Park, 2013[118]	182	Stroke/TIA patients	×	×	×	√	×	×	×	Posterior	BADE was associated with CMBs (OR 7.41, *P*=0.001).
Thijs, 2017[119]	3850	SIFAP1 study	√	√	√	√	×	×	×	Anterior Posterior	Patients with IADE more often had CMBs (16.3% vs. 4.7%, *P*=0.001), lacunes (29.1% v.s. 16.5%, *P*<*0*.001), more severe WMH (*P*<0.001), and lacunar stroke (18.1% vs. 12.4%, *P*=0.0018).
Del,	346	Communit	×	√	√	√	√	×	×	Posterior	BA dolichoectasia was associated with WMH (OR

2017[120]		y based									3.35, P=0.031) and deep CMBs (OR 4.78, P=0.001), but not with PVS and lacunes.
Zhai, 2018[76]	1237	Community based	×	√	√	√	√	√	×	Anterior Posterior	BADE was associated with BG-PVS (OR 2.20, P=.01), marginally with lacunes (OR 1.98, P=0.05) and CMBs (OR 2.02, P=0.07).
Zhang, 2018[121]	1089	CHANCE	√	×	×	×	×	×	×	Posterior	Lacunar stroke was more prevalent in patients with BADE than those without (29.6% vs. 22.7%, HR 2.87, P<0.001).
Gutierrez, 2019[122]	941	Community based	×	×	√	×	√	×	×	Anterior	MCA/ACA diameter was associated with PVS (β=0.177, P=0.002), but not with WMH.
Zhang, 2019[123]	212	Vertigo patients	×	√	√	×	×	×	×	Posterior	BA length (P<0.001) and tortuosity (P=0.001) related to WMH severity, but not with lacunes.
Yin, 2021[124]	469	Stroke patients	×	√	√	×	×	×	×	Anterior Posterior	IADE was associated with multi-lacuna (OR 2.14, P=0.015) and WMH (OR 2.78, P=0.001).
Del, 2021[125]	200	Stroke patients	√	×	×	×	×	×	×	Anterior Posterior	There was a trend towards higher IADE prevalence in those with lacunar stroke (27.6% vs. 17.6%, OR=1.8).
Osama, 2022[126]	200	Stroke patients	×	×	×	×	√	×	×	Posterior	CMBs were more common in patients with BADE than those without (84% vs. 53%, P=0.009).
Thiankhaw, 2025[127]	138	ICH patients	×	√	√	√	√	×	×	Anterior Posterior	IADE was associated with deep lacunes (OR 3.1, P=0.04), WMH (OR 3.29, P=0.04), deep CMBs (OR 2.8, P=0.04), but not with PVS.
Current study	229	Mild stroke patients	√	√	√	√	√	√	√	Anterior Posterior	BADE and arterial widening were associated with all cSVD markers apart from brain atrophy.

* Any new infarct detected on follow-up MRI.

†Refers to whether the investigated artery is located in the anterior or posterior circulation.

Abbreviations: ACA, anterior cerebral artery; BA, basilar artery; BADE, basilar artery dolichoectasia; BG, basal ganglia; CHANCE, Clopidogrel in High-risk patients with Acute Non-disabling Cerebrovascular Events Trial; CMB, cerebral microbleeds; cSVD, cerebral small vessel disease; CVD, cerebrovascular disease; HR, hazard ratio; IADE, intracranial artery dolichoectasia; ICH, intracranial hemorrhage; LS, lacunar stroke; MCA, middle cerebral artery; OR, odds ratio; PVS, perivascular spaces; SIFAP1, Stroke in Young Fabry Patients; WMH, white matter hyperintensity.

Supplemental Figures and Figure Legends

Figure S1. Illustration of severe stenosis in the left middle cerebral artery.

Intracranial artery stenosis was primarily assessed on arterial phase dynamic contrast-enhanced MRI, when stenosis was suspected, structural sequences were reviewed for confirmation. Panels A-D show consecutive sagittal slices from a dynamic contrast-enhanced MRI scan, progressing from the central (A) to peripheral slices (D), with red arrows indicating the site of stenosis. Panels E and F display axial T2-weighted images of the same artery.

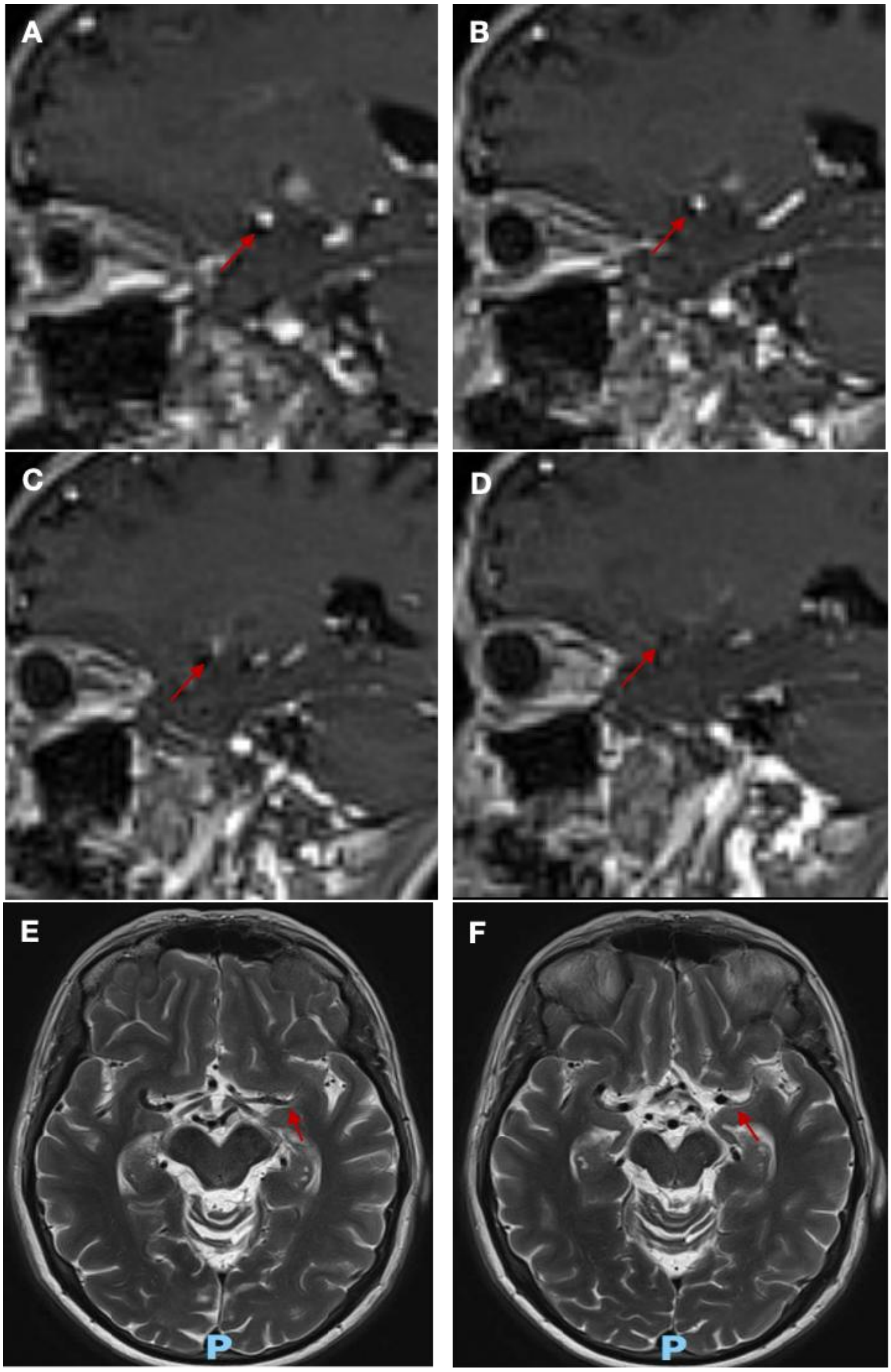

Figure S2. Assessment of intracranial artery dolichoectasia.

(A) Measurement of the bilateral internal carotid artery diameters at the vertical cavernous segment, and the diameter of the basilar artery at the midpontine level on axial T2-weighted imaging. (B) Measurement of the bilateral middle cerebral artery diameters at the M1 segment on axial T2-weighted imaging. (C) Grading of basilar artery lateral displacement using a validated 4-point grading scale: 0=at/below dorsum sellae, 1=within suprasellar cistern, 2=at the level of third ventricle floor, 3=indenting and elevating third ventricle floor. (D) Grading of basilar artery bifurcation height in the sagittal plane on T1-weighted imaging: 0=midline, 1=medial to clivus or dorsum sellae margin, 2=lateral to this margin, 3=cerebellopontine angle cistern.

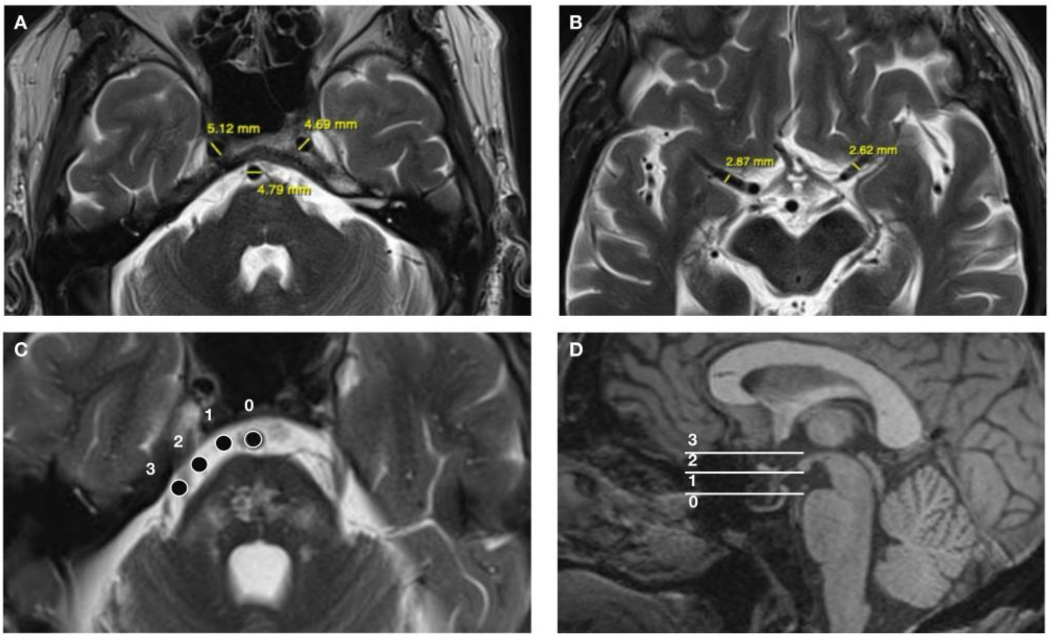

Figure S3. Distributions and anatomical features of incident infarcts.

(A) Distribution of subtypes among 130 incident infarcts in the per-infarct analysis, stratified by the presence or absence of large artery stenosis (LAS), any potential embolic source, and basilar artery dolichoectasia (*P* values from Rao-Scott cluster-adjusted chi square tests).

(B) Distribution of anatomical locations of 97 subcortical incident infarcts in the per-infarct analysis, stratified by the presence of LAS.

(C) Territorial concordance between incident infarcts and upstream stenosis among 26 infarcts in 10 patients with LAS (*P* value from Rao-Scott cluster-adjusted chi square tests).

Abbreviations: LAS, large artery stenosis; BADE, basilar artery dolichoectasia.

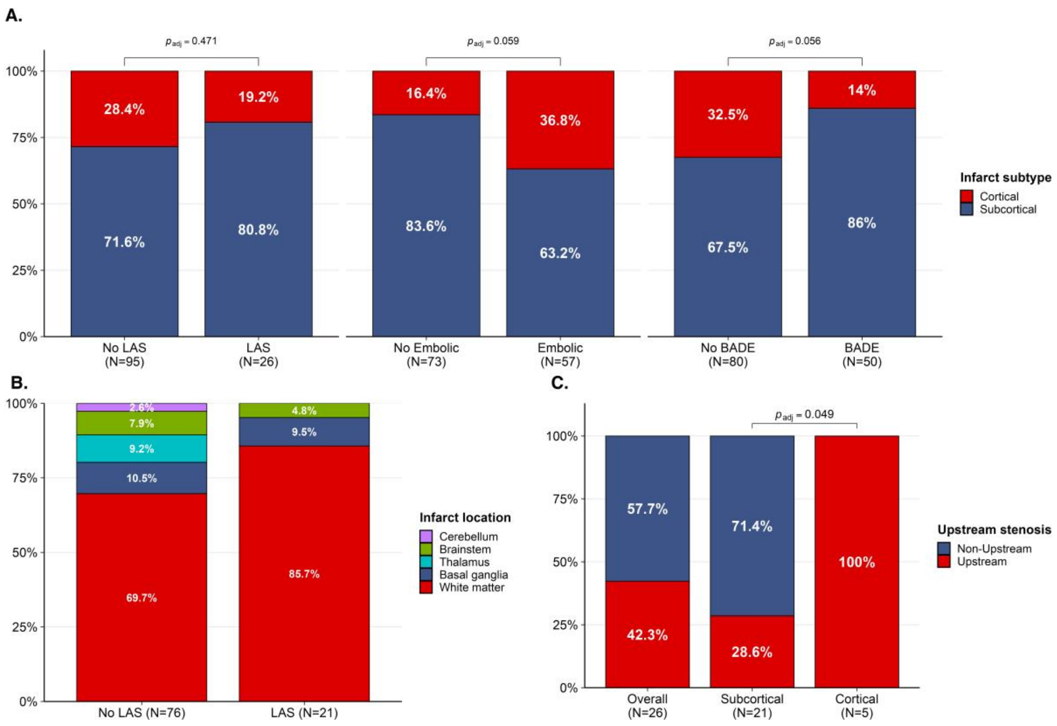

Figure S4. PRISMA diagram for systematic review.

(A) Associations between large artery stenosis and side-specific occurrence of lacunar stroke

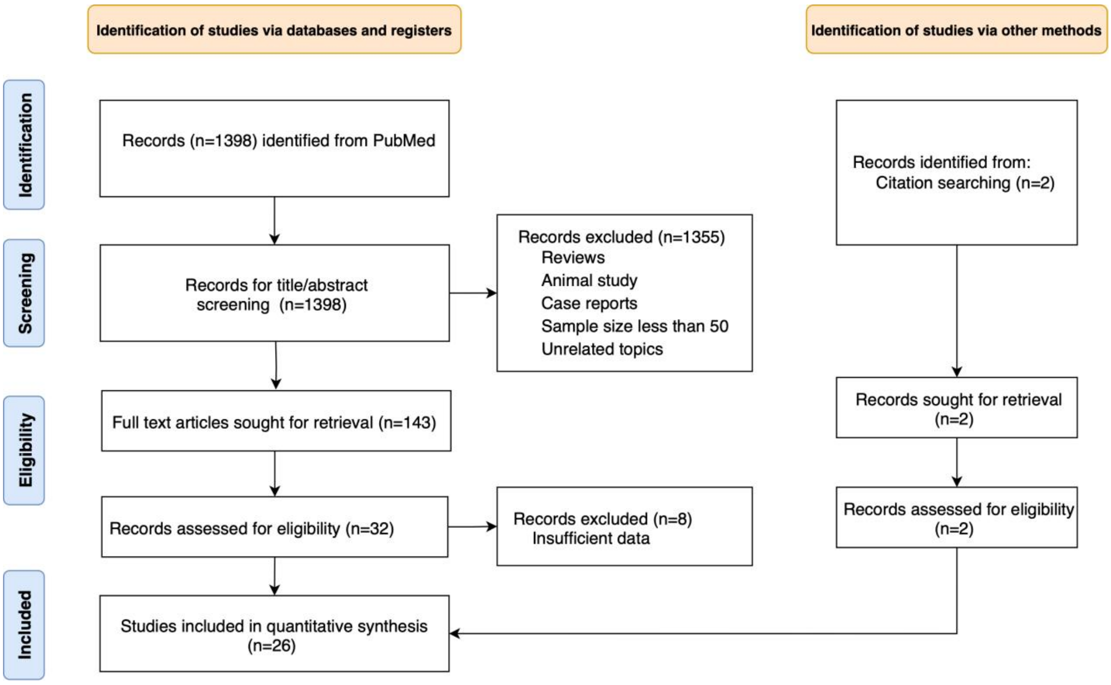

(B) Associations between large artery stenosis and cerebral small vessel disease (silent brain infarcts/lacunes, white matter lesions, cerebral microbleeds and perivascular spaces)

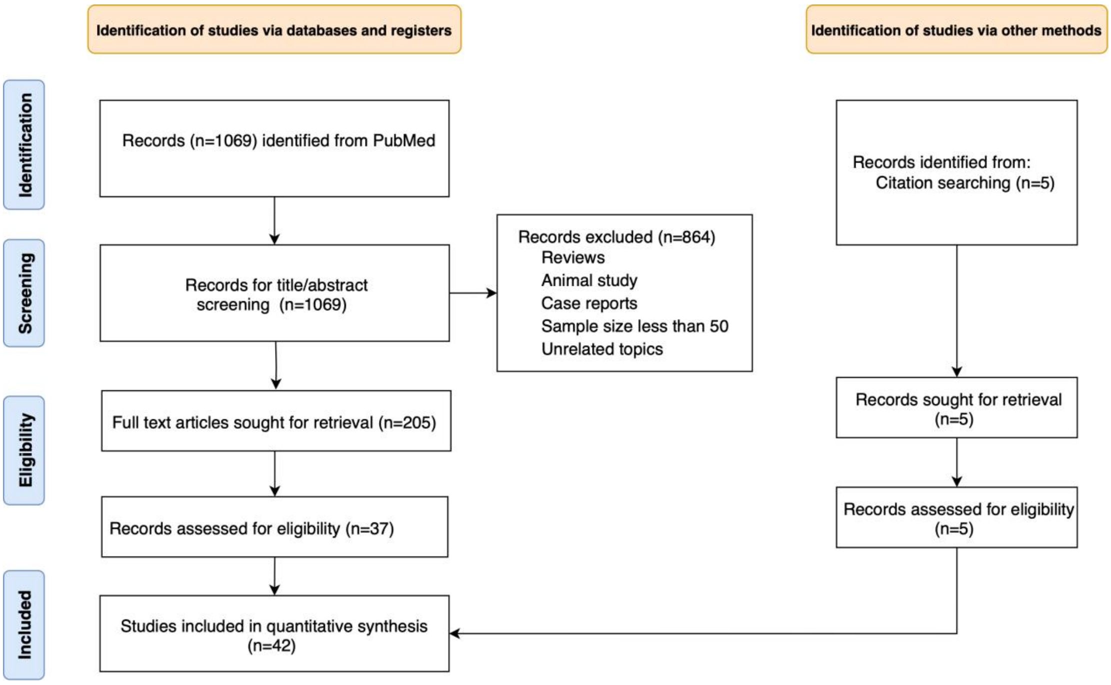

(C) Question: Associations between dolichoectasia and cerebral small vessel disease

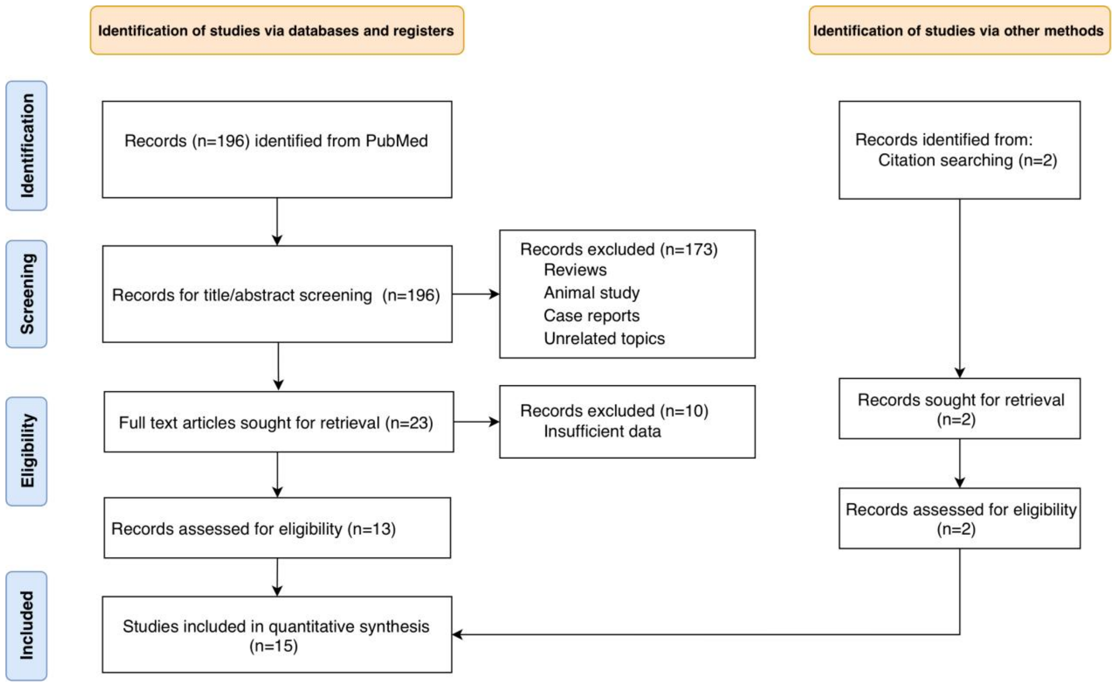

Figure S5. Meta-analysis of the prevalence and laterality of large artery stenosis in patients with lacunar stroke.

(A) Pooled prevalence of ipsilateral large artery stenosis in patients with lacunar stroke.

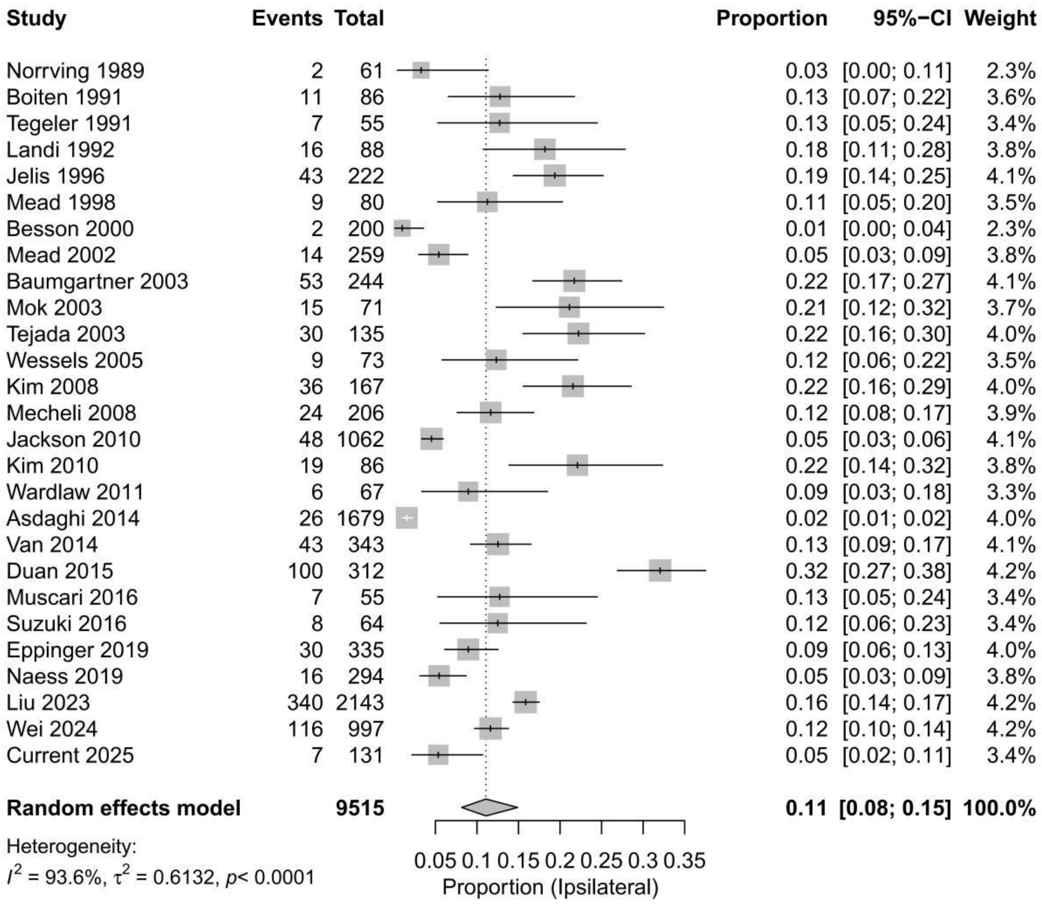

(B) Subgroup analysis: extracranial ipsilateral stenosis.

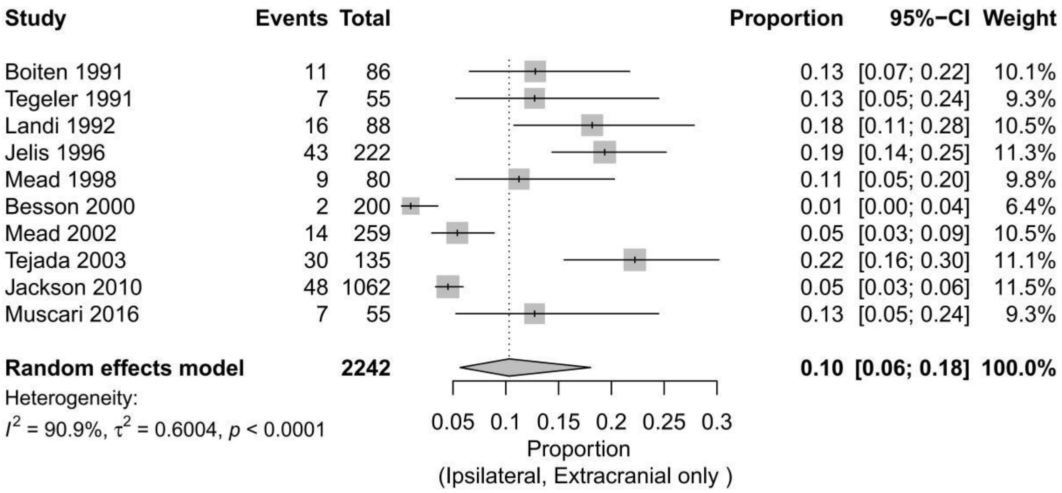

(C) Subgroup analysis: intracranial ipsilateral stenosis.

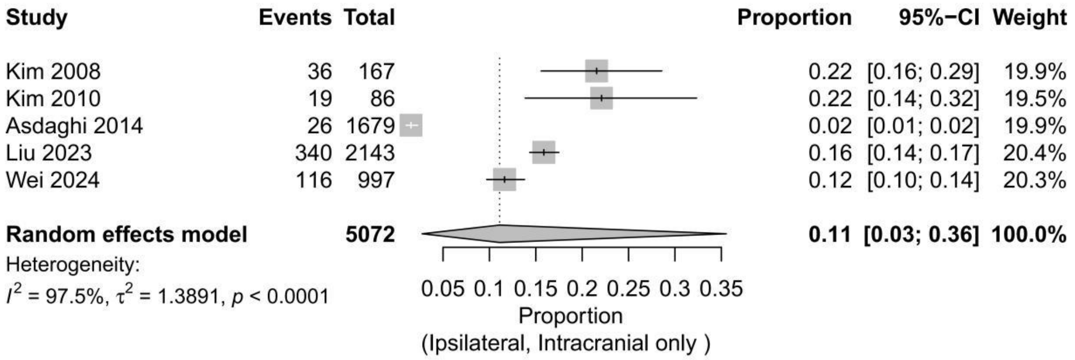

(D) Subgroup analysis: ipsilateral stenosis in Caucasian patients.

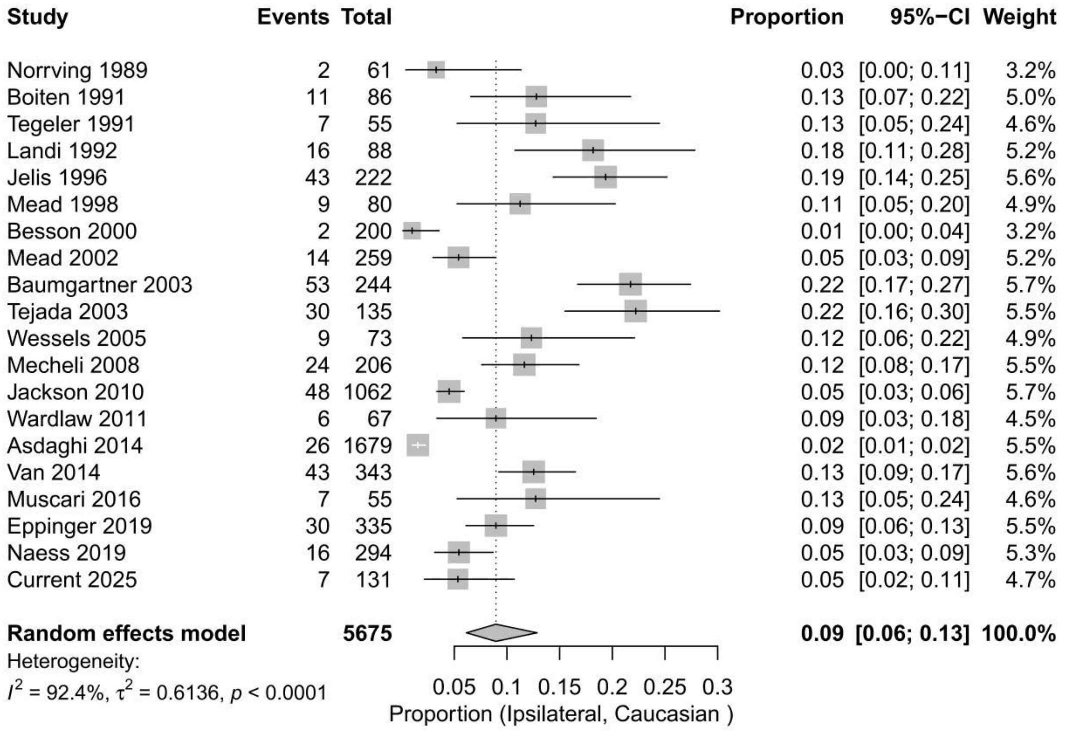

(E) Subgroup analysis: ipsilateral stenosis in Asian patients.

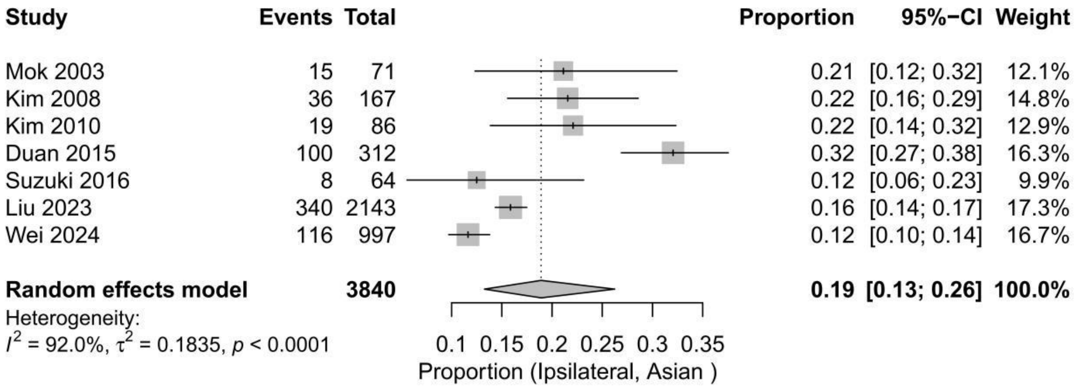

(F) Forest plot showing the risk difference (RD) in the prevalence of large artery stenosis between the ipsilateral and contralateral sides among patients with lacunar stroke. Each study contributed paired data, and the difference in prevalence (ipsilateral minus contralateral) was pooled using a random-effects meta-analysis. The pooled RD was 0.01 (95% CI: -0.04 to 0.06). Heterogeneity was substantial ($I^2 = 83.6\%$, $P < 0.0001$).

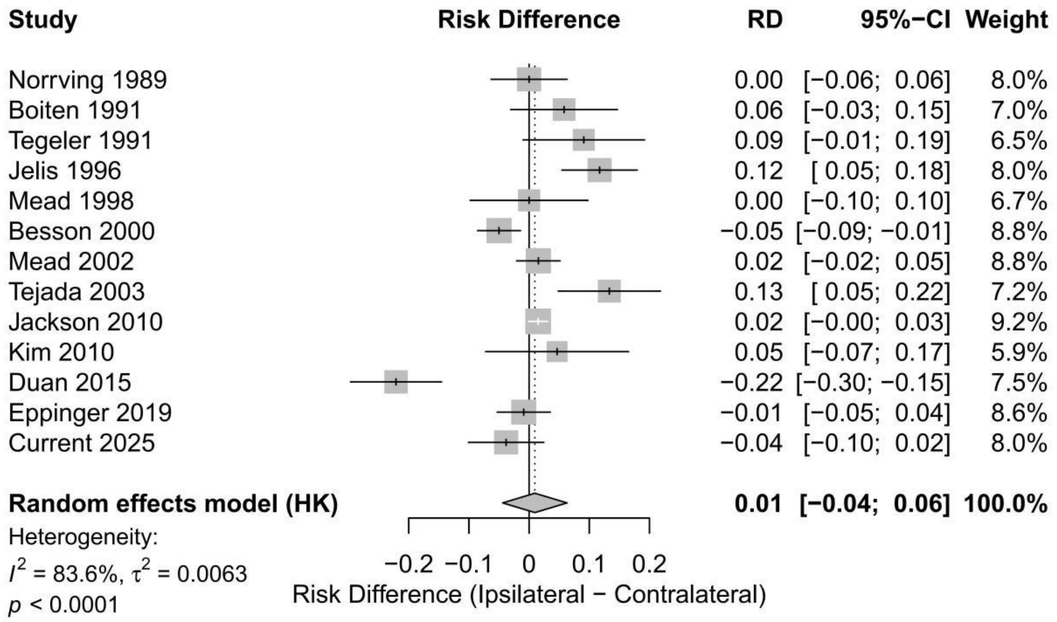

Figure S6. Sensitivity analyses of the pooled prevalence and risk difference estimates after exclusion of the study reporting the lowest prevalence.

(A) Pooled prevalence of ipsilateral large artery stenosis in patients with lacunar stroke.

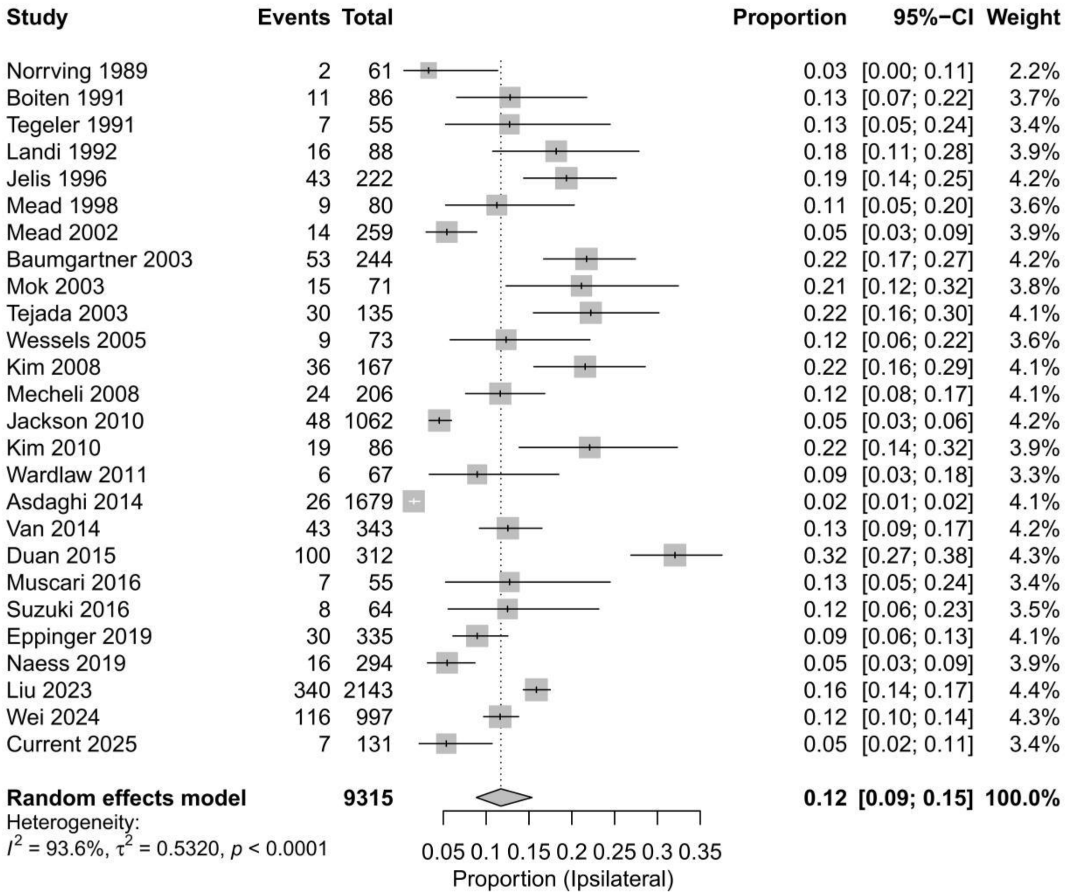

(B) Forest plot showing the risk difference (RD) in the prevalence of large artery stenosis between the ipsilateral and contralateral sides among patients with lacunar stroke.

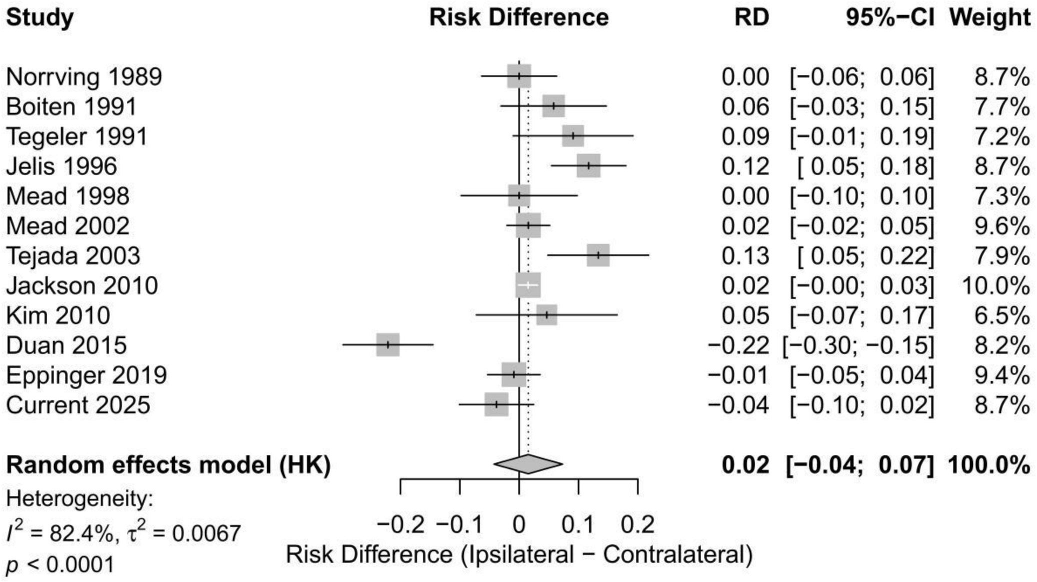

Figure S7. Meta-analysis of the association between dolichoectasia and cerebral small vessel disease. (A) Lacunar stroke, (B) Lacune, (C) Cerebral microbleed, (D) White matter hyperintensity, and (E) Perivascular spaces. Abbreviations: IADE, intracranial artery dolichoectasia; BADE, basilar artery dolichoectasia.

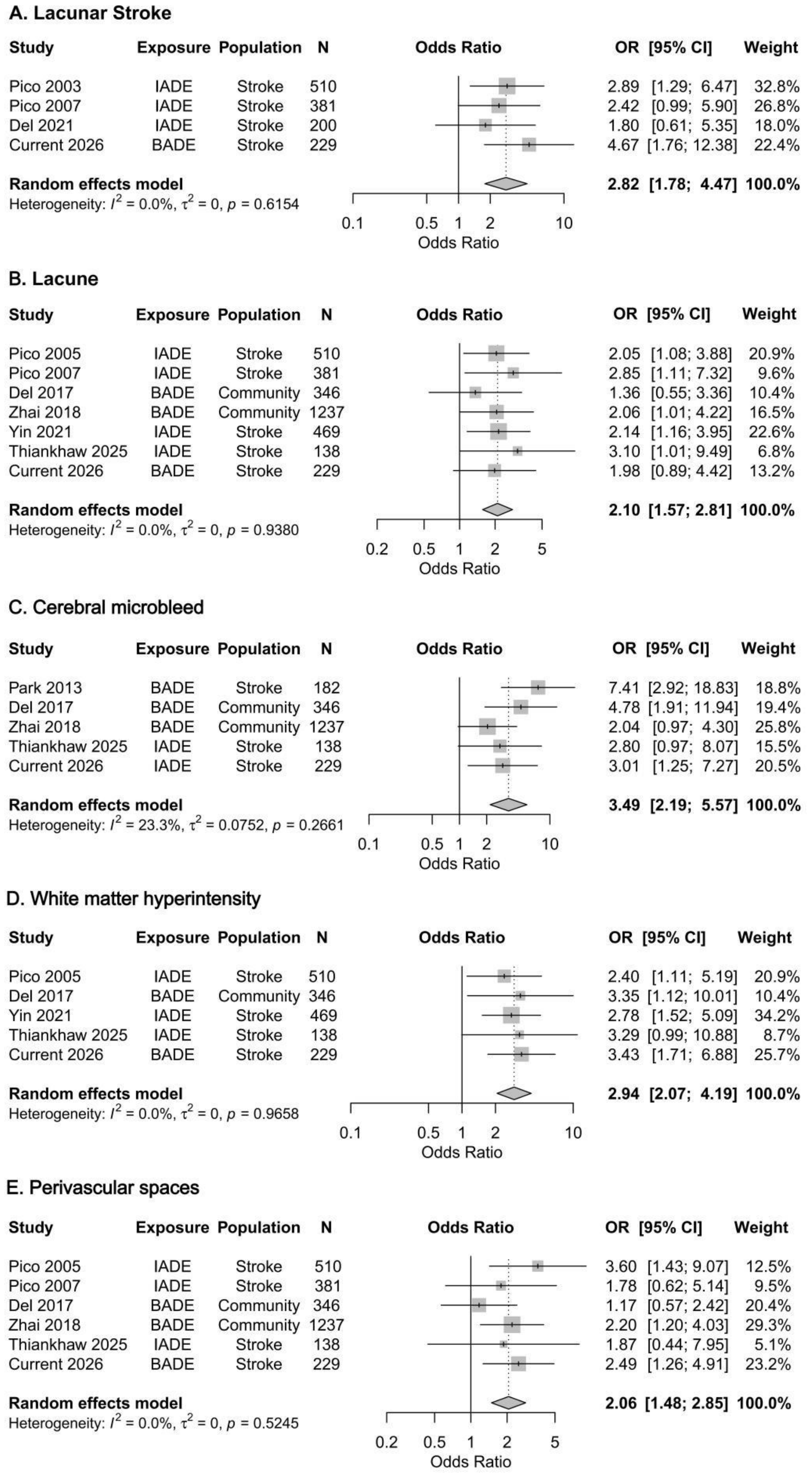

STROBE Checklist

	Item	Recommendation	Page
**Title and abstract**	1	(*a*) Indicate the study's design with a commonly used term in the title or the abstract	1-4
		(*b*) Provide in the abstract an informative and balanced summary of what was done and what was found	3-4
**Introduction**			
Background/rationale	2	Explain the scientific background and rationale for the investigation being reported	7
Objectives	3	State specific objectives, including any prespecified hypotheses	7-8
**Methods**			
Study design	4	Present key elements of study design early in the paper	8
Setting	5	Describe the setting, locations, and relevant dates, including periods of recruitment, exposure, follow-up, and data collection	8
Participants	6	(*a*) Give the eligibility criteria, and the sources and methods of selection of participants. Describe methods of follow-up	8
		(*b*) For matched studies, give matching criteria and number of exposed and unexposed	NA
Variables	7	Clearly define all outcomes, exposures, predictors, potential confounders, and effect modifiers. Give diagnostic criteria, if applicable	8-12
Data sources/ measurement	8*	For each variable of interest, give sources of data and details of methods of assessment (measurement). Describe comparability of assessment methods if there is more than one group	8-12
Bias	9	Describe any efforts to address potential sources of bias	14
Study size	10	Explain how the study size was arrived at	8
Quantitative variables	11	Explain how quantitative variables were handled in the analyses. If applicable, describe which groupings were chosen and why	8-12
Statistical methods	12	(*a*) Describe all statistical methods, including those used to control for confounding	12-13
		(*b*) Describe any methods used to examine subgroups and interactions	14
		(*c*) Explain how missing data were addressed	12-13
		(*d*) If applicable, explain how loss to follow-up was addressed	NA

		(*e*) Describe any sensitivity analyses	14
**Results**			
Participants	13*	(a) Report numbers of individuals at each stage of study—eg numbers potentially eligible, examined for eligibility, confirmed eligible, included in the study, completing follow-up, and analysed	14 Figure1
		(b) Give reasons for non-participation at each stage	Figure1
		(c) Consider use of a flow diagram	Figure1
Descriptive data	14*	(a) Give characteristics of study participants (eg demographic, clinical, social) and information on exposures and potential confounders	14
		(b) Indicate number of participants with missing data for each variable of interest	14
		(c) Summarise follow-up time (eg, average and total amount)	14
Outcome data	15*	Report numbers of outcome events or summary measures over time	14
Main results	16	(*a*) Give unadjusted estimates and, if applicable, confounder-adjusted estimates and their precision (eg, 95% confidence interval). Make clear which confounders were adjusted for and why they were included	14-18
		(*b*) Report category boundaries when continuous variables were categorized	14-18
		(*c*) If relevant, consider translating estimates of relative risk into absolute risk for a meaningful time period	14-18
Other analyses	17	Report other analyses done—eg analyses of subgroups and interactions, and sensitivity analyses	17
**Discussion**			
Key results	18	Summarise key results with reference to study objectives	18
Limitations	19	Discuss limitations of the study, taking into account sources of potential bias or imprecision. Discuss both direction and magnitude of any potential bias	22-23
Interpretation	20	Give a cautious overall interpretation of results considering objectives, limitations, multiplicity of analyses, results from similar studies, and other relevant evidence	18-22
Generalisability	21	Discuss the generalisability (external validity) of the study results	22
**Other information**			

Funding	22	Give the source of funding and the role of the funders for the present study and, if applicable, for the original study on which the present article is based	23-24
